# GASDERMIN D-mediated pyroptosis as a therapeutic target in TAU-dependent frontotemporal dementia mouse model

**DOI:** 10.1186/s12929-025-01210-1

**Published:** 2026-01-05

**Authors:** Ignacio Silva-Llanes, Lilia A. Smith, Aaron Abdelkader-Guillén, José Jiménez-Villegas, David Sarrió, Gema Moreno-Bueno, Isabel Lastres-Becker

**Affiliations:** 1 Instituto de Investigaciones Biomédicas “Sols-Morreale” CSIC-UAM, C/ Arturo Duperier, 4, 28029 Madrid, Spain; 2 Instituto de Investigación Sanitaria La Paz (IdiPaz), Madrid, Spain; 3 Translational Cancer Research Group, Area 3 Cancer, Instituto Ramón y Cajal de Investigación Sanitaria (IRYCIS), 28034 Madrid, Spain; 4 Centro de Investigación Biomédica en Red, Área Cáncer, CIBERONC, Instituto de Salud Carlos III, 28029 Madrid, Spain; 5 Fundación MD Anderson Internacional, 28033 Madrid, Spain; 6 Centro de Investigación Biomédica en Red, Área Enfermedades Neurodegenerativas, CIBERNED, Instituto de Salud Carlos III, 28029 Madrid, Spain

**Keywords:** TAU, Pyroptosis, NLRP3, Dimethyl fumarate (DMF), GASDERMIN D, Frontotemporal dementia (FTD), Alzheimer’s disease (AD)

## Abstract

**Background:**

Recent research has revealed a strong connection between neuroinflammation and TAU protein-related neurodegeneration. A key discovery shows that the NLRP3 inflammasome, when activated, can significantly impact TAU pathology and subsequent neuronal death. This process involves pyroptosis, a lytic form of programmed cell death driven by inflammasome activation, leading to GASDERMIN D (GSDMD) cleavage and the subsequent release of inflammatory molecules IL-1β and IL-18. In this study, we explore the role of pyroptosis and GSDMD in Alzheimer’s disease (AD) and tauopathy models, focusing on the TAU-induced neuroinflammatory process and its correlation with synaptic plasticity loss.

**Methods:**

Hippocampal tissue from AD patients at Braak stage II-III has been analyzed using qPCR to assess pyroptosis-related gene expression. To determine the role of TAU in pyroptosis and neuroinflammation, we used two different models: one based on intracerebral injection of an adeno-associated virus that specifically overexpresses TAU in the neurons of the hippocampus (AAV-TAU^P301L^), and a transgenic mouse model Tg-TAU^P301S^ at 8 and 10 months of age. Gene expression, protein levels, and neuroinflammation markers were evaluated using qPCR and immunofluorescence. Additionally, both genetic (GSDMD-deficient mice) and pharmacological (dimethyl fumarate, DMF) interventions targeting pyroptosis have been explored to assess their impact on neuroinflammation and synaptic plasticity.

**Results:**

AD patients exhibited increased expression of pyroptosis-related genes, supporting the involvement of pyroptosis in neurodegeneration. Furthermore, TAU overexpression induced pyroptosis in both mouse models, and GSDMD protein levels increased alongside reactive microglial morphology. Our data supports that TAU-induced neuroinflammation correlated with synaptic plasticity impairment. GSDMD deficiency significantly reduced pyroptosis-related markers associated to TAU, but unexpectedly worsened synaptic plasticity deficits, suggesting GSDMD may play a dual role in inflammation and synaptic function. Finally, we showed that DMF treatment suppressed pyroptosis gene expression, reduced GSDMD levels, and alleviated neuroinflammation, correlating with improved synaptic marker expression.

**Conclusion:**

Our findings demonstrate that TAU-induced pyroptosis contributes to neuroinflammation and synaptic dysfunction. While GSDMD inhibition mitigates inflammation, its absence exacerbates synaptic impairment, highlighting its complex role in tauopathies. Our results indicate that DMF treatment could offer a promising therapeutic avenue to modulate pyroptosis and neuroinflammation, and restore synaptic integrity in tauopathies.

**Supplementary Information:**

The online version contains supplementary material available at 10.1186/s12929-025-01210-1.

## Background

The TAU protein is a microtubule-associated protein (MAP), and its alterations are a defining feature of several brain diseases collectively known as tauopathies. These disorders include Alzheimer’s disease (AD), frontotemporal lobar degeneration (FTLD-TAU), progressive supranuclear palsy (PSP), and corticobasal degeneration, among others [1–3]. Mutations in the *MAPT* gene, which codes for TAU, are associated with inherited forms of FTLD, with examples like TAU^P301L^ and TAU^P301S^. These mutations weaken the ability of TAU to bind microtubules and promote the formation of abnormal protein filaments [3, 4]. In other tauopathies, such as AD, TAU malfunctions due to modifications like hyperphosphorylation, leading to toxic neurofibrillary tangles that impair neurons. Regardless of the underlying cause, tauopathies commonly involve synaptic dysfunction, neuronal loss, abnormal protein build-up, and neuroinflammation [5–7]. Recent insights highlight that cell death plays a pivotal role in driving inflammation. Emerging evidence points to a complex interplay between TAU pathology and inflammatory responses, fostering a harmful cycle sustained by activated microglia and injured neurons [8, 9].

The fact that the neuroinflammatory process is one of the earliest events associated with the progression of neurodegeneration, and that it further amplifies the neurodegenerative process, creating a vicious cycle that self-sustains and perpetuates neurodegeneration, indicates an intricate connection between these two processes [10, 11]. Moreover, it is also important to consider that recent evidence suggests glial cells, which actively participate in immune responses in the central nervous system (CNS), are a crucial third component of the synapse. Alongside pre- and post-synaptic neurons, these cells actively contribute to neurotransmission, neuronal excitability, and various forms of synaptic plasticity [12]. Thus, in various tauopathies, a process of microgliosis and astrogliosis has been described, accompanied by an increase in the expression of pro-inflammatory factors [8, 11, 13–15]. Recently, it has been reported that during this pro-inflammatory process, TAU induces the activation and formation of the NLRP3-dependent inflammasome [16–22]. While all the inflammasome sensors NLRP1, NLRP3, NLRC4, AIM2, and pyrin have the capability to form canonical inflammasome complexes, the NLRP3 is the best studied in neurological disorders, being directly involved in pyroptotic neuronal death [23]. Pyroptosis is a form of pro-inflammatory programmed cell death that can be activated in multiple ways. In the canonical pathway, following inflammasome assembly, CASPASE-1 is activated, and on the one hand, cleaves the executor protein GASDERMIN D (GSDMD). The released GSDMD N-terminal domain (NTD) oligomerizes and forms pores at the cell membrane, through which inflammatory molecules are secreted and finally leading to cell rupture [24–27]. On the other hand, the activation of CASPASE-1 also cleaves the precursors of IL-1β and IL-18 to produce their mature forms. These are released through the pores formed by the NTD GSDMD, resulting in pyroptotic cell death and the subsequent response of the immune system [28]. It is important to mention that activation of the NLRP3 inflammasome can lead to, but does not necessarily entail, pyroptosis. The cell can activate CASPASE-1, process IL-1β and IL-18, and even release these cytokines without undergoing cell death, depending on the cellular context, levels of active GSDMD, and other regulatory signals [29]. Research has shown that NLRP3 inflammasome-driven neuronal pyroptosis is strongly linked to the progression of several neurodegenerative disorders [30–33]. The implication of pyroptosis in the neurodegenerative process of Alzheimer’s disease (AD) has been evidenced in studies conducted on peripheral blood mononuclear cells extracted from AD patients [33], as well as in studies using microarray analysis followed by differential gene expression analysis of hippocampal samples from AD patients [34] and in CA1 and the temporal cortex [35]. However, the involvement of pyroptosis in TAU-induced neurodegeneration and whether its modulation affects the neurodegenerative process has not been studied until now. To date, regarding the TAU protein, all studies have focused on the NLRP3-dependent inflammasome [17, 19, 36, 37], but it has not been investigated whether GSDMD is activated or involved in TAU-dependent pathological processes. Therefore, in this study, we tackled this challenge by first analyzing the role of TAU in the pyroptosis process. Furthermore, we aimed to determine the involvement of GSDMD in TAU-associated neuroinflammation and neurodegeneration, by using GSDMD-knockout mice. Finally, we investigated whether pharmacological modulation of GSDMD through dimethyl fumarate (DMF) treatment could serve as a novel therapeutic strategy for tauopathies [38]. Previous studies from our group had already described the neuroprotective effect of DMF treatment [39], but not in the context of pyroptosis modulation. DMF, besides inhibiting GSDMD, is also an activator of the Nuclear Factor erythroid-derived 2-like 2 (NRF2) transcription factor. NRF2 has become essential in reducing neurodegeneration by influencing processes such as proteostasis, oxidative stress, and neuroinflammation. Normally, NRF2 levels are kept low through various regulatory mechanisms. The main process controlling NRF2’s transcriptional activity involves its interaction with the E3 ligase adapter, Kelch-like ECH-associated protein 1 (KEAP1). This binding allows NRF2 to be presented for ubiquitination by the Cullin 3 and RING-box protein 1 (CUL3/RBX1) complex, leading to its degradation by the proteasome [40–44]. However, when key cysteine residues in KEAP1 are modified by electrophiles, like DMF, or reactive oxygen species (ROS), conformational changes occur, preventing KEAP1 from targeting NRF2 for degradation. As a result, NRF2 accumulates and translocates to the nucleus, where it binds to an enhancer sequence called the antioxidant response element (ARE) in the promoter regions of NRF2 target genes [45]. In collaboration with members of the small musculo-aponeurotic fibrosarcoma (MAF) family, NRF2 recruits additional components of the transcriptional machinery. Another mechanism for regulating NRF2 involves the E3 ligase adapter β-transducin repeat-containing E3 ubiquitin-protein ligase (β-TrCP), which presents NRF2 to a CUL1/RBX1 complex [46, 47], leading to an alternative pathway for NRF2’s ubiquitin-dependent proteasomal degradation. Our work explores the therapeutic potential of DMF as a multifactorial modulator of the TAU-associated neurodegenerative process.

## Methods

### Human tissues

All human samples and clinical data included in this study were provided by the Biobank Banco de Tejidos CIEN (PT17/0015/0014), integrated in the Spanish National Biobanks Network and they were processed following standard operating procedures with the appropriate approval of the Ethics and Scientific Committees. Immediately after brain extraction, midsagittal sectioning was performed to separate the right and left hemispheres of the brain. The right hemisphere was sliced and quick frozen fresh at − 50 °C (in NOVEC) and were immediately placed at − 80 °C, for storage.

The frozen post-mortem hippocampal tissues were obtained from four control and four AD patients (Braak stages II-III) within less than 6 h post-mortem interval (See more information at Supplementary Table S1), according to the standardized Biobank procedures. These frozen samples were used for RNA and qPCR analysis. The protocol used was similar to the one described in [48].

### Animals and stereotaxic injections

Wild-type C57BL/6 J mice were bred and housed (three to four mice per cage) in temperature-controlled cages (~ 23 °C) under a 12/12 h light/dark cycle with free access to water and standard chow in the Instituto de Investigaciones Biomédicas “Sols-Morreale” core. These animals come from the production colony of the animal facility at the Instituto de Investigaciones Biomédicas “Sols-Morreale”. For the adeno-associated viral model (AAV-TAU^P301L^), 6-month-old animals were used [48–52]. Viral vector injections were performed under ketamine/xylazine anesthesia (8 mg/kg ketamine and 1.2 mg/kg xylazine) on adult mice. Surgery was performed using a stereotaxic frame (RWD Life Science, USA) and a 5 µl Hamilton syringe fitted with a pulled glass capillary tube (outer diameter of 60–80 µm). Recombinant adeno-associated viral vectors of serotype 9, which express the human protein TAU^P301L^ under control of the synapsin 1 gene promoter (AAV-TAU^P301L^), were injected in the right hippocampus (ipsilateral side) as described elsewhere [39, 48, 49]. The overexpressed isoform is microtubule-associated protein TAU isoform 2 (NP_005901). Briefly, 2 μL of AAV suspension containing 3.5 × 10^12^ vp/mL were injected at the stereotaxic coordinates − 2.00 mm posterior, − 1.5 mm lateral, and -1.8 mm ventral relative to bregma, and the left hippocampus (contralateral side) was used as a control. A control AAV9 that expressed enhanced green fluorescent protein (EGFP) (AAV9-EGFP) did not have any significant effect on neuron viability, gliosis, or inflammation (data not shown). A similar procedure was employed on 6-month-old *Gsdmd*-deficient mice (C57BL/6N-*Gsdmd*^*em4Fcw*^/J; JAX:032410, The Jackson Laboratory) and wild-type littermates, with experimental procedure explained in detail in Fig. 5A. 8 and 10-month-old transgenic mice overexpressing hTAU^P301S^ protein (B6;C3-Tg(Prnp-MAPT*P301S)PS19Vle/J, The Jackson Laboratory) were used. Tg-TAU^P301S^ (PS19) carried a mutant (P301S) human microtubule-associated protein tau (*MAPT*) gene driven by the mouse prion-protein promoter (*Prnp*). These animals showed progressively accumulated TAU in association with striking neuron loss as well as hippocampal and entorhinal cortical atrophy by 8–12 months of age [53]. For treatment, DMF (100 mg/kg) (Sigma-Aldrich) was suspended in 0.8% methocel (Sigma-Aldrich) and given by oral gavage for 21 days (AAV-TAU^P301L^-model; experimental procedure explained in detail in Fig. 8A) or for 45 days every other day (Tg-TAU^P301S^-model; experimental procedure explained in detail in Fig. 10A). We did not detect significant weight loss, hair loss or other gross alterations in the DMF-treated mice either. All experiments were performed in a P2 biosafety facility and by certified researchers according to regional, national, and European regulations concerning animal welfare and animal experimentation, and were authorized by the Ethics Committee for Research of the Universidad Autónoma de Madrid with Ref PROEX 130.4/21 and PROEX 153.2/24. Every effort was taken to minimize the number of animals used and their suffering. To estimate the appropriated sample size, we employed G*Power (Heinrich Heine Universität Düsseldorf). The total number of mice used in the whole study was: (i) AAV-TAU^P301L^ n = 12; (ii) 8-month-old Tg-TAU^P301S^ (n = 5 WT, n = 7 Tg-TAU^P301S^); (iii) 10-month-old Tg-TAU^P301S^ (n = 5 WT, n = 5 Tg-TAU^P301S^) (iv) *Gsdmd*-deficient mice (n = 12 *Gsdmd*^+*/*+^, n = 10 *Gsdmd*^*−/−*^); (vi) AAV-TAU^P301L^ + DMF (n = 11 WT-VEH; n = 10 WT-DMF); (vii) 6 ^1/2^-month-old Tg-TAU^P301S^ + DMF (n = 6 WT-VEH; n = 6 WT-DMF; n = 6 Tg-TAU^P301S^-VEH; n = 6 Tg-TAU^P301S^-DMF). In our prior work with this experimental model, we have not detected significant differences between male and female subjects [48–52]. Consequently, our experimental groups included mice of both sexes, paired in equal numbers. Nevertheless, we consistently monitored potential sex-based differences throughout all experiments. Our observations confirmed that no significant disparities between sexes were present in the results of this study.

### Randomization and blinding

Animals were randomized for treatment, and data collection and evaluation of all experiments were conducted blind to group identity. Data and statistical analyses were performed in accordance with recommended guidelines for experimental design and analysis in pharmacology [54].

### Analysis of mRNA levels by quantitative real-time PCR

The animals that were to be used for brain dissection were sacrificed by cervical dislocation. Immediately afterward, the brain was extracted, and under a magnifying glass, both hippocampi were dissected separately to differentiate the control hippocampus (contralateral) from the one where TAU^P301L^ is overexpressed (ipsilateral). All these procedures were performed on ice. Once dissected, the tissues were frozen in dry ice and stored at − 80 °C until further processing. For both human or mouse samples, total RNA extraction, reverse transcription, and analysis of mRNA levels by quantitative real time PCR (qPCR) was performed as described in a previous article [48]. One microgram of RNA from each sample was treated with DNase (Invitrogen) and reverse-transcribed using high-capacity RNA-to-cDNA Master Mix (Applied Biosystems, REF. 4388950). Primer sequences are presented in Supplementary Table S2. Data analysis was based on the 2^−ΔΔCT^ method with normalization of the raw data by the geometric mean of the housekeeping genes *Actb*, *Gapdh* and *Tbp* (NZYtech). All PCRs were performed in triplicate by using the QuantStudio3 from Applied Biosystems.

### Immunoblotting

Whole-tissue lysates were prepared in RIPA buffer (25 mM Tris–HCl pH 7.6, 150 mM NaCl, 1 mM EGTA, 1% Igepal, 1% sodium deoxycholate, 0.1% SDS, 1 mM PMSF, 1 mM Na3VO4, 1 mM NaF, 1 μg/mL aprotinin, 1 μg/mL leupeptin and 1 μg/mL pepstatin). Whole-cell lysates containing 50 μg of whole proteins from hippocampi were loaded for SDS-PAGE electrophoresis. Immunoblots were performed as described in [48]. The primary antibody used for GSDMD detection is described in Supplementary Table S3.

### Immunohistochemistry on mouse tissues

Mouse brain tissue was sectioned at 30 µm on a cryostat (Leica CM 1950) and stained as free-floating sections with Netwell baskets. As previously described [48], a standard avidin–biotin immunohistochemical protocol was used. Primary and secondary antibodies are described in Supplementary Table S2. Briefly, the primary antibody used was anti-human TAU (TAU -HT-7), for 24 h at RT. After washing, the sections were first incubated with the secondary biotinylated antibody and then with the ABC kit system to increase sensitivity, and developed using 3, 3-diaminobenzidine (DAB). Finally, tissues were dehydrated in ethanol and cleared in xylene. Sections were mounted with DEPEX and coverslipped. The immunohistochemistry images were captured using a directed Axiophot microscope (Zeiss) with transmitted light and epifluorescence. It has a camera with DP70 color with a DP Controller image capture system. The objectives that we used were: ACHROPLAN 4 × and Plan-NEOFLUAR 10x/0.3. Mice that did not show correct hTAU expression were discarded from further analysis (data not shown).

### Immunofluorescence on mouse tissues

Immunofluorescence assays were performed on 30-µm thick coronal brain sections. The protocol followed was previously described [49]. Primary and secondary antibodies are described in Supplementary Table S3. Images were taken on the confocal Leica Stellaris 8 TauSTED (SEMOC, Instituto de Investigaciones Biomédicas “Sols-Morreale”). The mean intensity was analyzed at the CA3 area (contralateral vs. ipsilateral or Tg-TAU^P301S^ vs. WT) from mice by the Image J1.54d program. A total of 3 images per side and condition were analyzed as follows. The images were transformed into 16-bit with the Image J program. Then, with the “Free Hand Selection” tool of the Imagen J 1.54d program, we manually selected the area of each image.

### Stereological analysis of microgliosis and astrogliosis

Cell counts were performed using Fiji Software (http://fiji.sc/Fiji). For each mouse, 2–3 hippocampal sections were used for analysis [55]. The error coefficient attributable to the sampling was calculated according to Gundersen and Jensen (1987), and values ≤ 0.10 were accepted. (n = 5–6 animals per experimental group). The colocalisation analysis was based on measuring the fluorescence intensity detected in each of the pixels of the analysed images, using the Intensity Correlation Analysis complement of Fiji software (http://fiji.sc/Fiji) as a reference, as well as the values of Pearson’s correlation coefficient [56, 57]. The fluorescence values of each channel in each pixel of the image were represented on the same graph to obtain an intensity profile that reflected the colocalisation of the proteins of interest (GraphPad Prism 10, San Diego, CA).

### Morphological analysis of microglial cells

To analyse the cell size and the morphological structure of IBA1^+^ cells with Fiji software, we followed the previously described protocol [58]. Briefly, the images were transformed into 8-bit format, and the threshold was adjusted to delimit the perimeter of the cells. *Unsharp Mask* and *Despeckle* tools were used to sharpen the images and remove unspecific spots in the background, respectively. With the *Analyze Particles* tool, the areas of each individual cell were determined to calculate the average cell size and circularity, a parameter representing the degree of roundness of cells. The same images were used to determine the number of branches and the average length of the branches with the *Skeletonize* and *Analyze skeleton* tools. (n = 5–6 animals per experimental group).

### In silico search for NRF2 binding sites

The script used in this study, with some modifications, was previously described [59]. Briefly, it uses as input a Browser Extensible Data (BED) file containing the ChIP-seq peaks for the transcription factors of analysis, a text file containing a list of RefSeq transcripts accession numbers, and a position frequency matrix (PFM) file from the JASPAR database containing the consensus transcription factor binding sites to be computed. Additionally, it makes use of the BED file at the UCSC Genome Browser, Table Browser resource (https://genome.ucsc.edu/cgi-bin/hgTables) containing the locations of every transcript and its RefSeq accession number in the genome. For the overlapping with regulatory elements, a combined segmentation BED file was generated by concatenating, using BEDTools, the ENCODE Candidate Cis-Regulatory Elements (cCREs) [60] combined from all cell types for the mm10 mouse genome. The script retrieves the genomic coordinates for the desired transcripts, extends them 5000 bp upstream of the transcription start site, and intersects them with ChIP-seq peaks downloaded from all experiments in ChIP-Atlas [61] for the given transcription factors using the wrapper of BEDTools for Python, pybedtools [62, 63]. In this analysis, all the available binding sites for NFE2L2, MAFF, MAFK, MAFG, and BACH1 were downloaded and intersected with the extended transcripts of *Nlrp3*, *Asc*, *Casp1*, *Gsdmd*, *Il1b*, *Il18rap* and *Aim2* genes Table 1. Then, the sequences of the ChIP-seq peaks were extracted using pybedtools from the FASTA file of the mm10 mouse genome. The profile for NFE2L2 binding sites was downloaded from the JASPAR database [64] in PFM format from the entry MA0150.1. Absolute frequencies are turned into a PSSM (position specific-scoring matrix), containing scores through the log2 (odds-ratio) (odds ratio: observed frequency/expected frequency). One unit was added as a pseudo-count to each absolute frequency to avoid log(0). Scoring of each site followed a similar procedure as we have previously described [65]. Briefly, a sliding window of a width dependent on the profile to be used was passed over the extracted sequences. Each nucleotide in the sliding window received a score according to the PSSM and then, the score from each nucleotide was added up in order to provide an absolute score for the site. The relative score, the maximal and minimal scores were obtained with a given PSSM and computed as (absolute score +|minScore|)/(|maxScore| +|minScore|). Sites with a relative score below 0.8 were discarded, and the remaining ones were provided as a BED file. In order to detect active regions, the script makes use of pybedtools to intersect the segmentation file with the regions described in the regulatory element bed file.

**Table 1:**
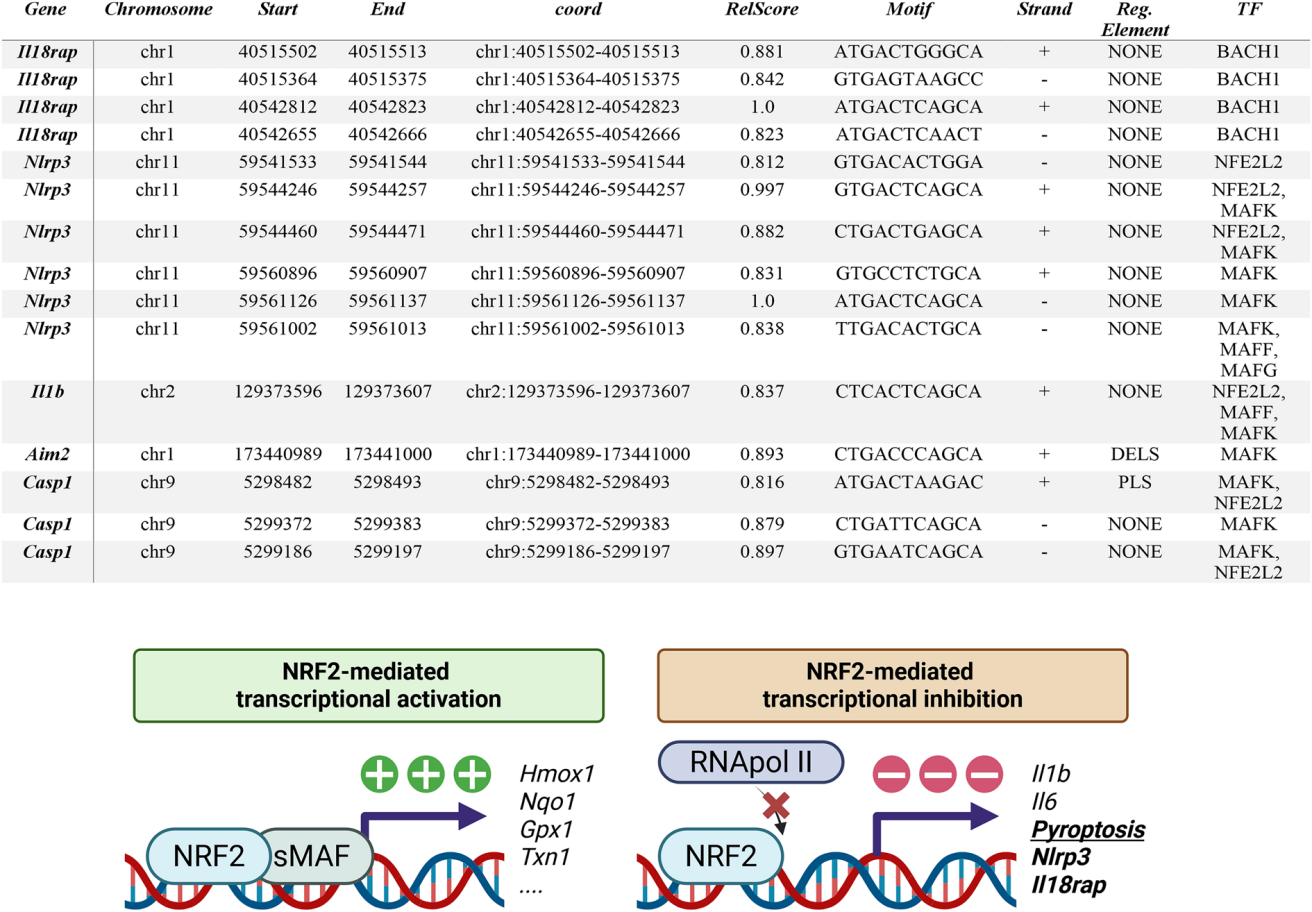
Putative ARE sequences identified in the mouse genome with a relative score over 80%

Columns 1 and 2 describe the genes containing the transcription factor-binding sites and their specific locations according to the GRCm38 (mm10) mouse genome assembly. The Motif column contains the specific sequence that was identified as a putative ARE sequence. Relative scores were calculated against the consensus binding sequence according to the position frequency matrix of the JASPAR database. The strand column indicates in which strand of the DNA, relative to the transcript sense is the putative ARE sequence: sense (+) or antisense (-). The Regulatory Element column provides the segmentation annotation from the ENCODE Candidate Cis-Regulatory Elements (cCREs) combined from all cell types at the UCSC Genome Browser. The TFs column indicates the transcription factor for which the ChIP-seq site belonged in the ChIP-Atlas database. *PLS, promoter-like signature; dELS; distal enhancer-like signature. Figures: Brief description of how NRF2 can induce or repress the expression of specific genes

### Statistical analysis

Data are presented as mean ± SEM (Standard Error of the Mean). To confirm which statistical test had to be used, we employed GraphPad InStat 3 including the analysis of the data to normal distribution via the Kolmogorov–Smirnov test. Furthermore, statistical assessments of differences between groups were analyzed (GraphPad Prism 10, San Diego, CA). Unpaired Student’s *t*-tests or two-way ANOVA with post hoc Bonferroni were used as appropriate.

## Results

### Increased expression of genes involved in the pyroptosis process in the hippocampus of Alzheimer’s disease patients

**Fig. 1:**
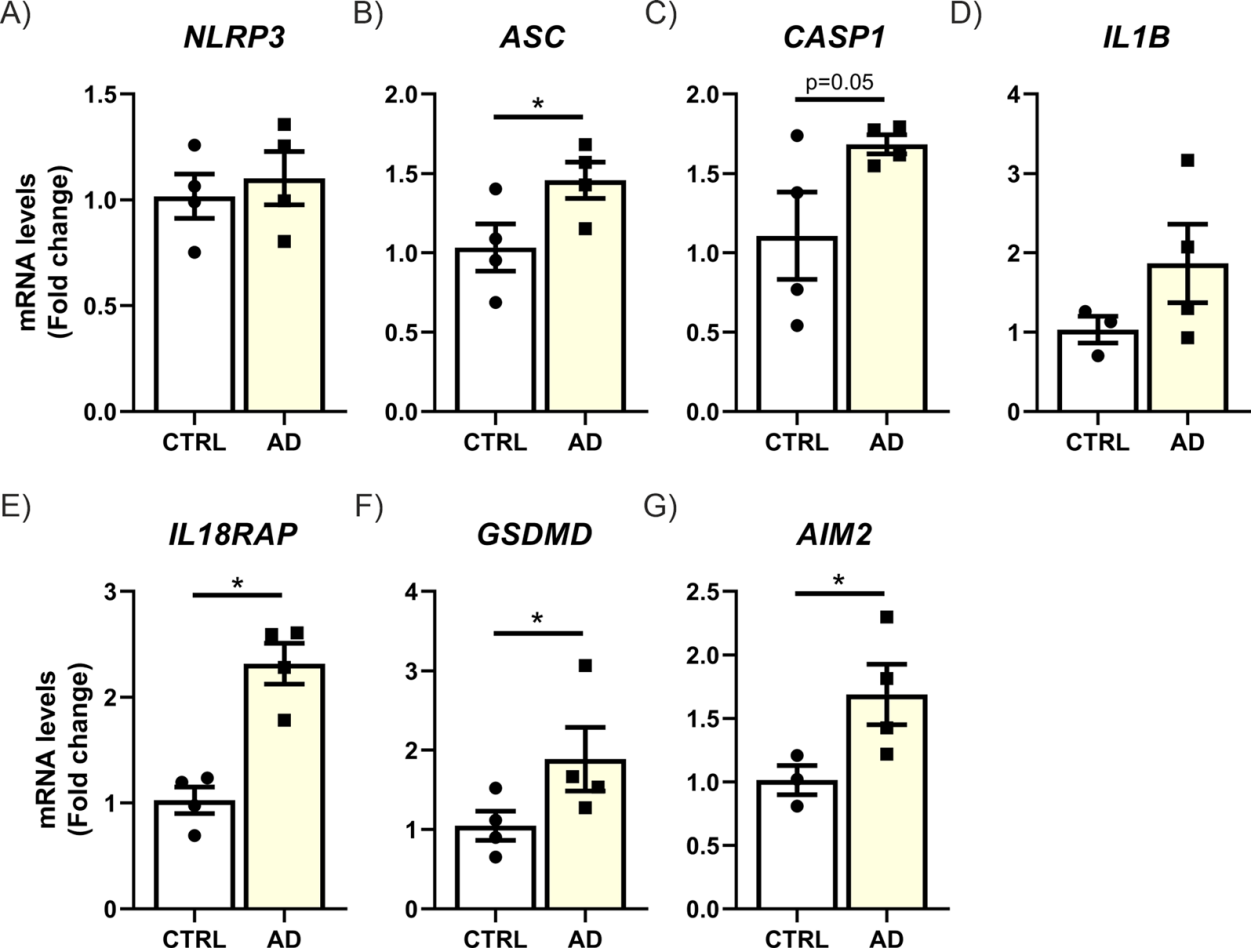
Increased mRNA expression of pyroptosis markers in hippocampus of AD patients. Quantification of mRNA levels of genes involved in the pyroptosis process in hippocampal samples from AD patient’s versus controls. Bars represent the mean of 3–4 samples ± SEM. Asterisks indicate significant differences of *p < 0.05 comparing experimental groups by t-Student’s *t*-test followed by Mann–Whitney post-test

We analyzed the expression of key genes involved in the pyroptosis process using qPCR in hippocampal samples from AD patients at Braak stage II-III. It is important to note that interleukin-18 receptor accessory protein (IL18RAP) is an essential subunit of the IL-18 receptor (IL-18R) complex, required for high-affinity IL-18 binding and effective signal transduction [66]. Previous studies by the group have shown that increases in *Il18rap* expression correlate more strongly with the pyroptosis process than even IL-18 levels themselves [67]. In Fig. 1, a statistically significant increase in mRNA levels of *ASC*, *IL18RAP*, *GSDMD*, and *AIM2* can be observed, while *CASP1* and *IL1B* show a trend toward increased levels in patients. No significant changes were detected for *NLRP3*. These data support the presence of pyroptosis in the neurodegenerative process of AD.

### TAU induces the pyroptosis process in different tauopathy mouse models

**Fig. 2:**
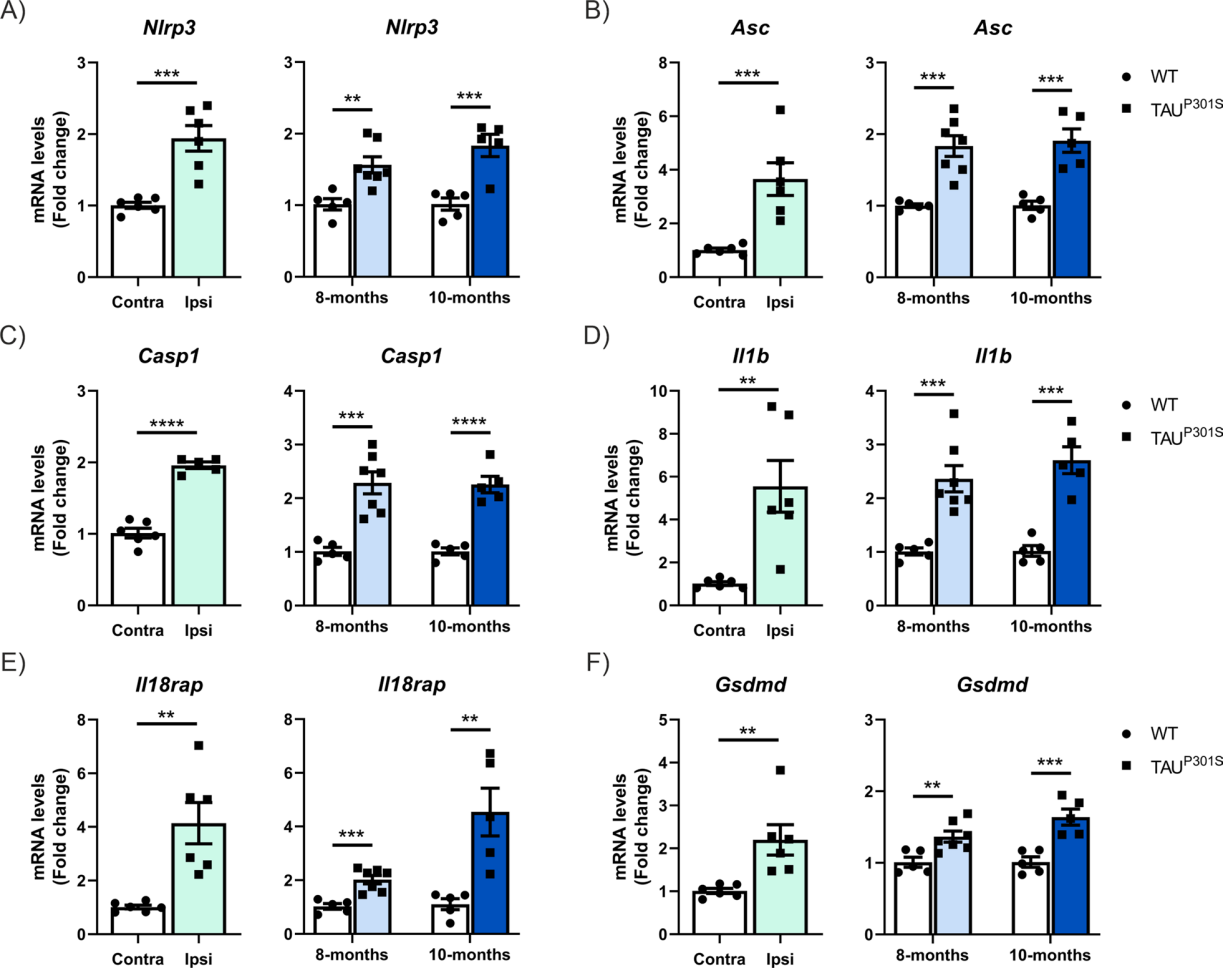
TAU overexpression induces the expression of pyroptosis markers in two different tauopathy mouse models. **A**–**F** Analysis of mRNA levels of genes involved in the pyroptosis process in hippocampal samples from AAV-TAU^P301L^ mice (light green) and in 8-month-old (light blue) and 10-month-old (dark blue) Tg-TAU^P301S^ mice

**Fig. 3:**
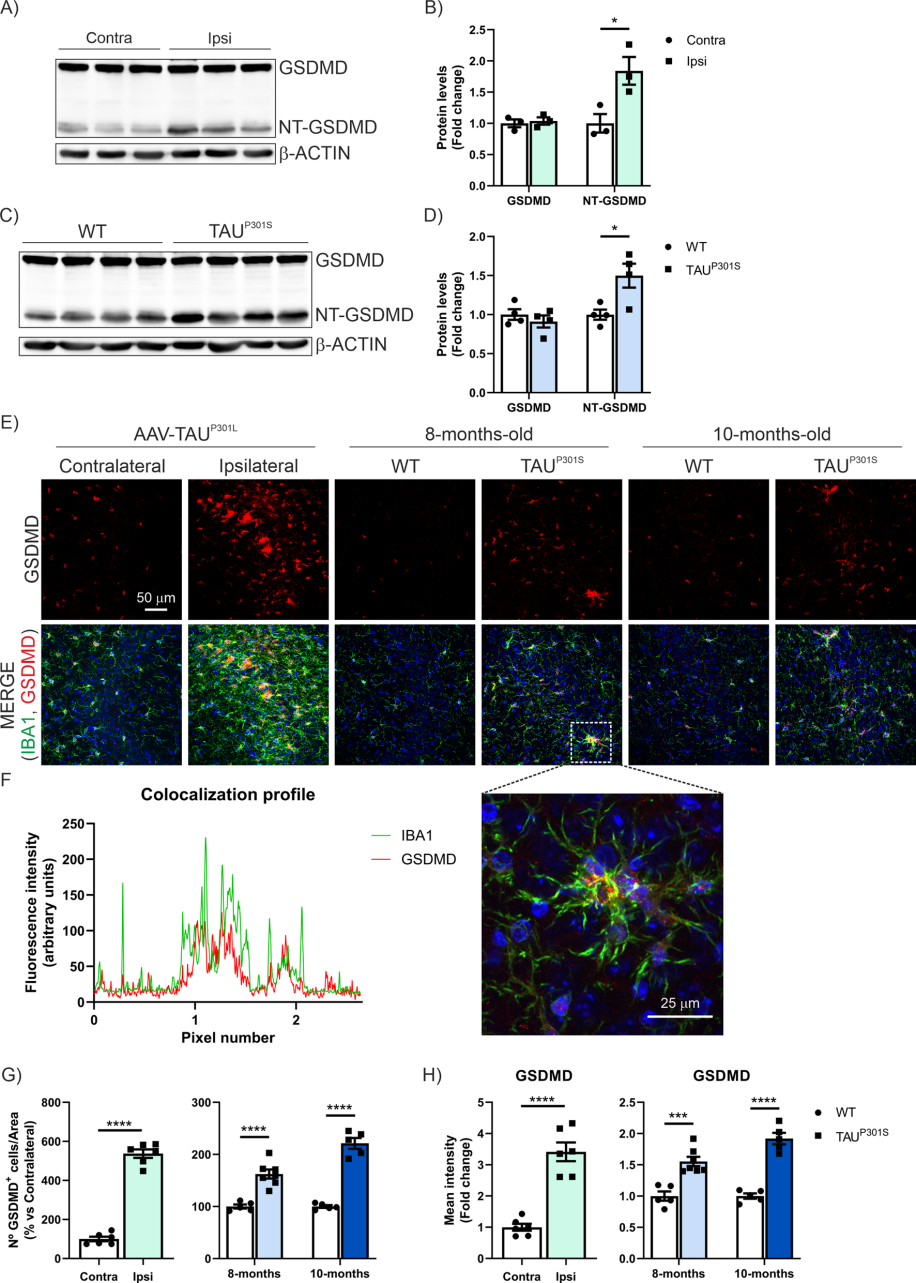
TAU overexpression induces the expression of NT-GDMD in two different tauopathy mouse models. Protein levels of GDMD and cleavage NT-GSDMD were analyzed by immunoblotting and their respective protein quantifications in hippocampal samples from (**A**, **B**) AAV-TAU^P301L^ mice (light green); n = 3 samples per experimental group ± SEM and (**C**, **D**) 8-month-old (light blue) Tg-TAU^P301S^ mice. (**E**) Immunostaining of GSDMD (red) and IBA1 (green) in the CA3 region of both tauopathy models. (**F**) Colocalization profile and sample. (**G**) Quantification of the number of GSDMD^+^ cells. (**H**) Quantification of GSDMD fluorescence intensity. Bars represent the mean of 5–7 samples ± SEM. Asterisks denote significant differences of **p < 0.01; ***p < 0.001; ****p < 0.0001 comparing each group by Student’s *t*-test

Considering that AD is a secondary tauopathy, we next aimed to determine the role of TAU in this process. To this end, we analyzed the pyroptosis process in the hippocampus of two different tauopathy mouse models. The first model is based on the neuronal overexpression of TAU^P301L^ in the hippocampus through AAV injection. The second model analyzed consists of Tg-TAU^P301S^ mice at 8 and 10 months of age. The analysis of mRNA levels of *Nlrp3*, *Asc*, *Casp1*, *Il1b*, *Il18rap*, *Gsdmd*, and *Aim2* by qPCR demonstrates a significant increase induced by TAU overexpression in both models (Fig. 2A–F and Supplementary Figure S1A, respectively). In the Tg-TAU^P301S^ model, we observe a slight increase in *Gsdmd* expression in 10-month-old mice compared to 8-month-old mice. To further investigate the implication of GSDMD, we analyzed its expression by Western-blot (WB) (whole hippocampus lysates) and immunofluorescence (IF) in the CA3 region of the hippocampus (Fig. 3). In hippocampal samples processed to obtain whole cell lysates for WB analysis, we observed that TAU overexpression leads to an increase in the p30 NT-GSDMD levels (Fig. 3A, B). Similar results were obtained in both mouse models (Fig. 3A–D). It is important to note that total GSDMD levels (p50 GSDMD) remain unchanged, indicating that the GSDMD induction caused by TAU overexpression is exclusively in the NT-GSDMD form. This point is crucial for the IF analysis. By IF, we stained microglial cells with the marker IBA1 and observed that the GSDMD-positive cells were microglia, as corroborated by the colocalization profile analysis (Fig. 3E–F). In this IF analysis, we observed that TAU overexpression significantly increases both the number of GSDMD^+^ cells (Fig. 3E-F) and the protein expression levels (Fig. 3E-G) in both the AAV-TAU^P301L^ model and the Tg-TAU^P301S^ mice. Moreover, in the Tg-TAU^P301S^ mice, this increase is proportional to the age of the mice. Based on the data obtained through WB, we can suggest that this increase corresponds to the NT-GSDMD form. Currently, there are no commercially available NT-GSDMD-specific antibodies for IF in mice. Our data also demonstrate that this process occurs in microglial cells, as GSDMD expression is observed specifically in IBA1^+^ cells. Furthermore, when microglial morphology shifts to a more reactive state, there is also an increase in GSDMD expression. It is important to highlight that this process is selective for GSDMD, as we have observed that, for example, *Gsdme* is not affected by TAU overexpression in either mouse model (Supplementary Figure S1B).

### Inverse correlation between neuroinflammation and synaptic plasticity in different tauopathy mouse models

It has been described that inflammatory responses triggered by microglial pyroptosis are linked to neurodegeneration and neuronal cell death [68]. Therefore, we next analyzed the neuroinflammatory process and whether there was an inverse correlation with the expression of synaptic plasticity markers in the CA3 region in both tauopathy models. Previous data from our group had already identified that TAU overexpression induced reactive astrogliosis in CA3 [48, 51, 52]. The occurrence of astrogliosis was confirmed using the astrocytic marker GFAP, both by IF and qPCR. As shown in Supplementary Figure S1C-D, TAU overexpression significantly increases both the number of astrocytes and induces morphological changes. This change is also reflected in an increased expression of *Gfap* mRNA levels (Supplementary Figure S1E) in the two models, and at both 8 and 10 months of age in Tg-TAU^P301S^ mice. Similarly, we analyzed the microgliosis process using the microglial marker IBA1. TAU overexpression also led to an increase in the number of microglial cells (Supplementary Figure S1F) and mRNA expression levels of *Iba1* (Supplementary Figure S1G) in both the AAV-TAU^P301L^ model and the Tg-TAU^P301S^ mice in the CA3 region. These data clearly highlight the connection between TAU and the neuroinflammatory process.

**Fig. 4:**
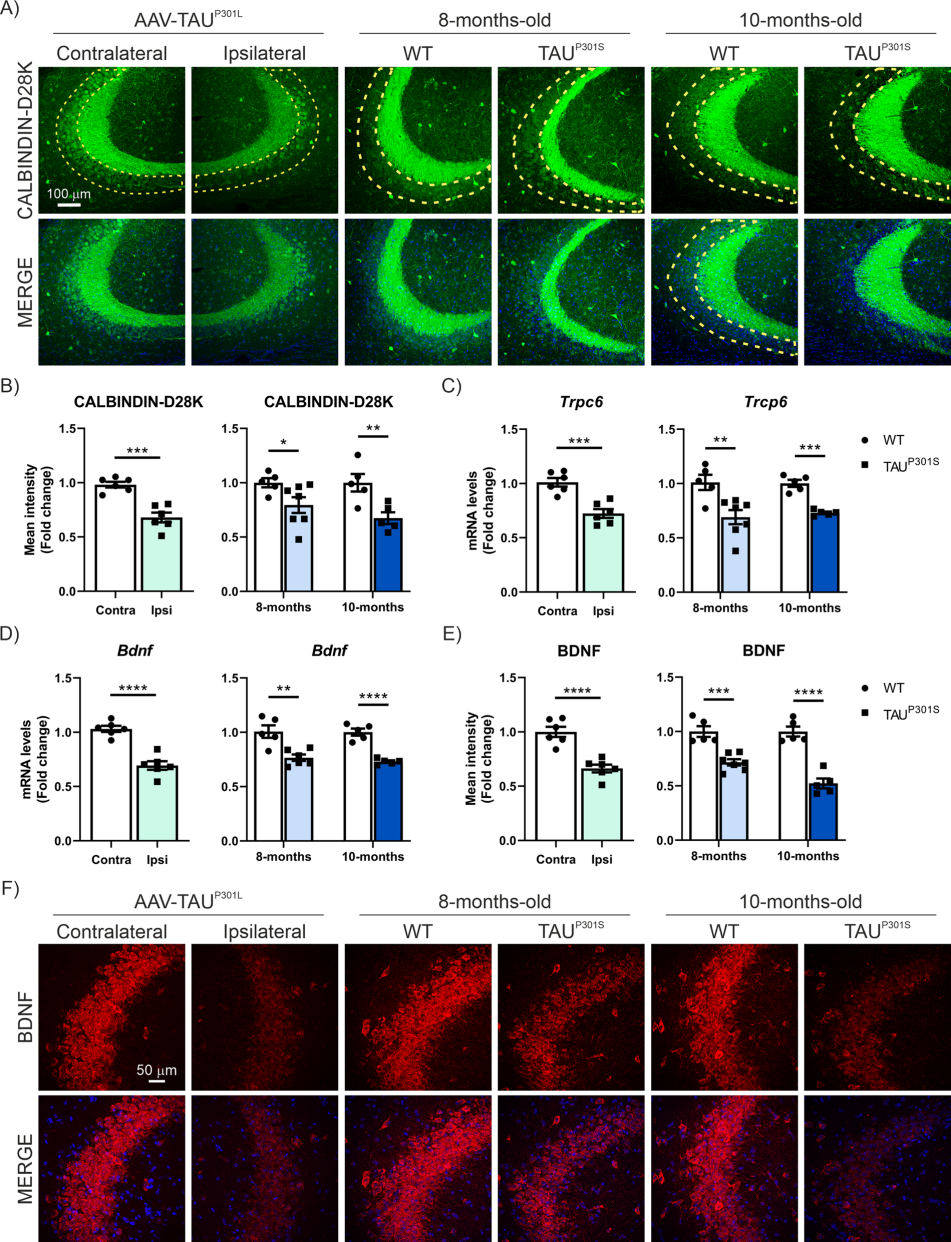
AAV-TAU^P301L^ and Tg-TAU^P301S^ mouse models show a decrease of synaptic plasticity markers in the hippocampus due to TAU overexpression.** A** Immunostaining of CALBINDIN-D28K in the CA3 region of FTD-TAU models. **B** Quantification of CALBINDIN-D28K fluorescence intensity in CA3 in AAV-TAU^P301L^ mice (light green) and in 8-month-old (light blue) and 10-month-old (dark blue) Tg-TAU^P301S^ mice. Analysis of (**C**) *Trcp6* and (**D**) *Bdnf* mRNA levels in both FTD-TAU models. **E** Quantification of BDNF fluorescence intensity. **F** Immunostaining of BDNF in the CA3 region. Bars represent the mean value of 5–7 samples ± SEM. Asterisks indicate significant differences of *p < 0.05; **p < 0.01; ***p < 0.001; ****p < 0.0001 comparing each group by Student’s *t*-test

On the other hand, growing evidence suggests that inflammation is intricately linked to TAU pathology and plays a role in its progression [19, 20, 69, 70]. Previously, our group described that TAU overexpression reduces the levels of CALBINDIN-D28K, a marker of synaptic plasticity, in the CA3 region [52]. These findings have been confirmed in the AAV-TAU^P301L^ model and also in Tg-TAU^P301S^ mice, with a more pronounced decrease observed in older mice (Fig. 4A, B). To further explore synaptic plasticity alterations, we also analyzed the mRNA expression levels of transient receptor potential canonical 6, *Trpc6* (Fig. 4C) and Brain derived neurotrophic factor, *Bdnf* (Fig. 4D). TRPC6 is associated with synapse function and contributes to hippocampal-dependent cognitive processes [71] and BDNF is a key molecule involved in synaptic plasticity changes [72]. In both markers, we observed a significant reduction in mRNA levels in the two models, the AAV-TAU^P301L^ and Tg-TAU^P301S^ mice. Additionally, through immunofluorescence analysis we confirmed further that TAU overexpression significantly reduces BDNF expression levels in the CA3 region (Figs. 4E, F). Taken together, our data suggest that TAU overexpression induces neuroinflammatory processes while inversely correlating with a reduction in the expression levels of synaptic plasticity markers.

### GSDMD-deficiency reduces the expression of proinflammatory markers associated with TAU overexpression

**Fig. 5:**
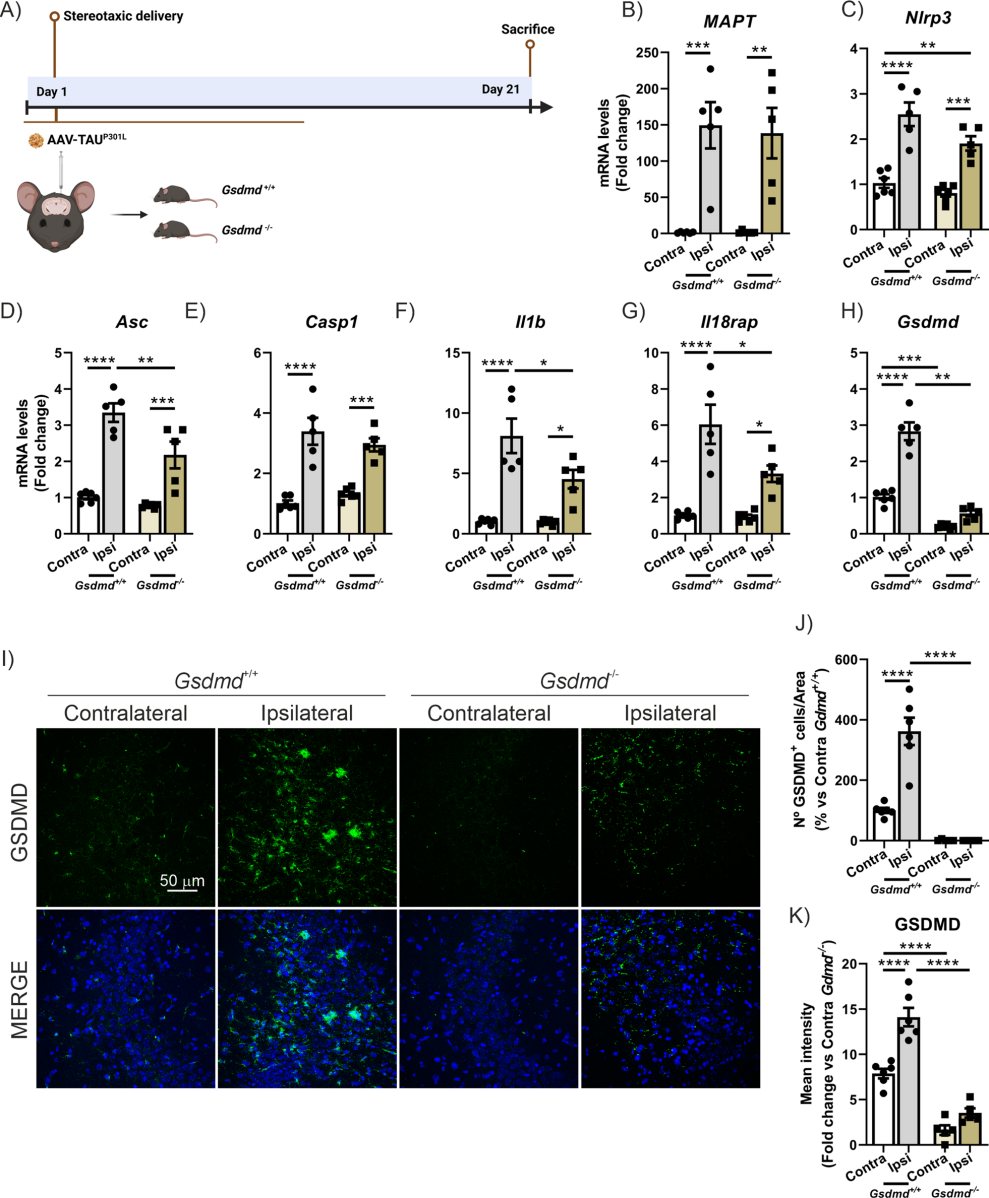
GSDMD deficiency slightly reduces the mRNA levels of pyroptosis markers in hippocampus. **A** Timeline representation of the experimental design: 6 months-old *Gsdmd*^+*/*+^ or *Gsdmd*^*−/−*^ mice were stereotaxic injected with AAV-TAU^P301L^ in the right hippocampus (ipsilateral side), and were sacrificed after 21 days. Analysis of mRNA levels of (**A**) *MAPT* and (**B**–**G**) genes involved in the pyroptosis process in the hippocampus of TAU^P301L^-overexpressing *Gsdmd*^+/+^ and *Gsdmd*^*−/−*^ mice. **H** Quantification of the number of GSDMD^+^ cells. **I** Quantification of GSDMD fluorescence intensity. **J** Immunostaining of GSDMD in the CA3 region. Bars represent the mean value of 4–5 samples ± SEM. Asterisks indicate significant differences of *p < 0.05; **p < 0.01; ***p < 0.001; ****p < 0.0001 comparing each group by two-factor ANOVA test followed by Bonferroni post-test

Next, we aimed to uncover the role of pyroptosis in the neurodegenerative process associated with TAU. To achieve this, we employed two different approaches. First, we used a genetic approach by utilizing GSDMD-deficient mice in the AAV-TAU^P301L^ -based tauopathy model. Second, we implemented a pharmacological approach by administering the GSDMD inhibitor, dimethyl fumarate (DMF), in both the AAV-TAU^P301L^ model (preventive study) and Tg-TAU^P301S^ mice (protective study). This dual approach allowed us to determine the specific role of GSDMD mediated pyroptosis in the neurodegenerative process induced by TAU. As shown in the experimental design in Fig. 5A, AAV-TAU^P301L^ was injected into 6-month-old *Gsdmd*-deficient and wild-type mice, which were sacrificed after 21 days. Before sacrificing the animals, a behavioral test was conducted to evaluate the impact of GSDMD deficiency and TAU overexpression on cognitive function (novel object recognition (NOR) test). On the habituation day (Day 1), an open field test was also performed to assess differences in locomotor activity among the mice. We observed that while there were no differences between Sham and Gsdmd^+/+^ mice, Gsdmd^−/−^ mice showed significantly reduced movement (data not shown). During the training phase, all groups explored both objects equally; however, Gsdmd^−/−^ mice exhibited a shorter exploration time, likely due to reduced locomotion, compared to Sham and Gsdmd^+/+^ mice, which showed no differences in exploratory behavior. On the test day, Gsdmd^−/−^ mice remained in the corners of the arena, making it impossible to carry out the analysis. The lack of movement in GSDMD-deficient mice prevented us from drawing any conclusions regarding their cognitive status following TAU overexpression. For this reason, no cognitive tests were included in this study.

At the molecular level, the deficiency in *Gsdmd* did not cause alterations in *MAPT* mRNA expression levels compared to wild-type mice (Fig. 5B). Regarding genes involved in the pyroptosis process, we observed that TAU overexpression in the context of GSDMD-deficiency, significantly reduced the levels of *Asc*, *Il1b*, *Il18rap* (Fig. 5D, F, and G) and *Aim2* (Supplementary Figure S2A), and a moderately decreased of *Nlrp3* and *Casp1* expression (Fig. 5C and E), albeit were not statistically significant, compared to WT mice. Additionally, as expected, upon TAU over-expression the levels of GSDMD at mRNA (Fig. 5H) and the proportion of GSDMD^+^ cells, by IF (Fig. 5I–K) strongly increased in WT mice, while minimal background signal was detected in GSDMD KO mice. Interestingly, TAU did not provoke any changes in *Gsdme* expression in neither WT nor GSDMD KO mice (Supplementary Figure S2B).

**Fig. 6:**
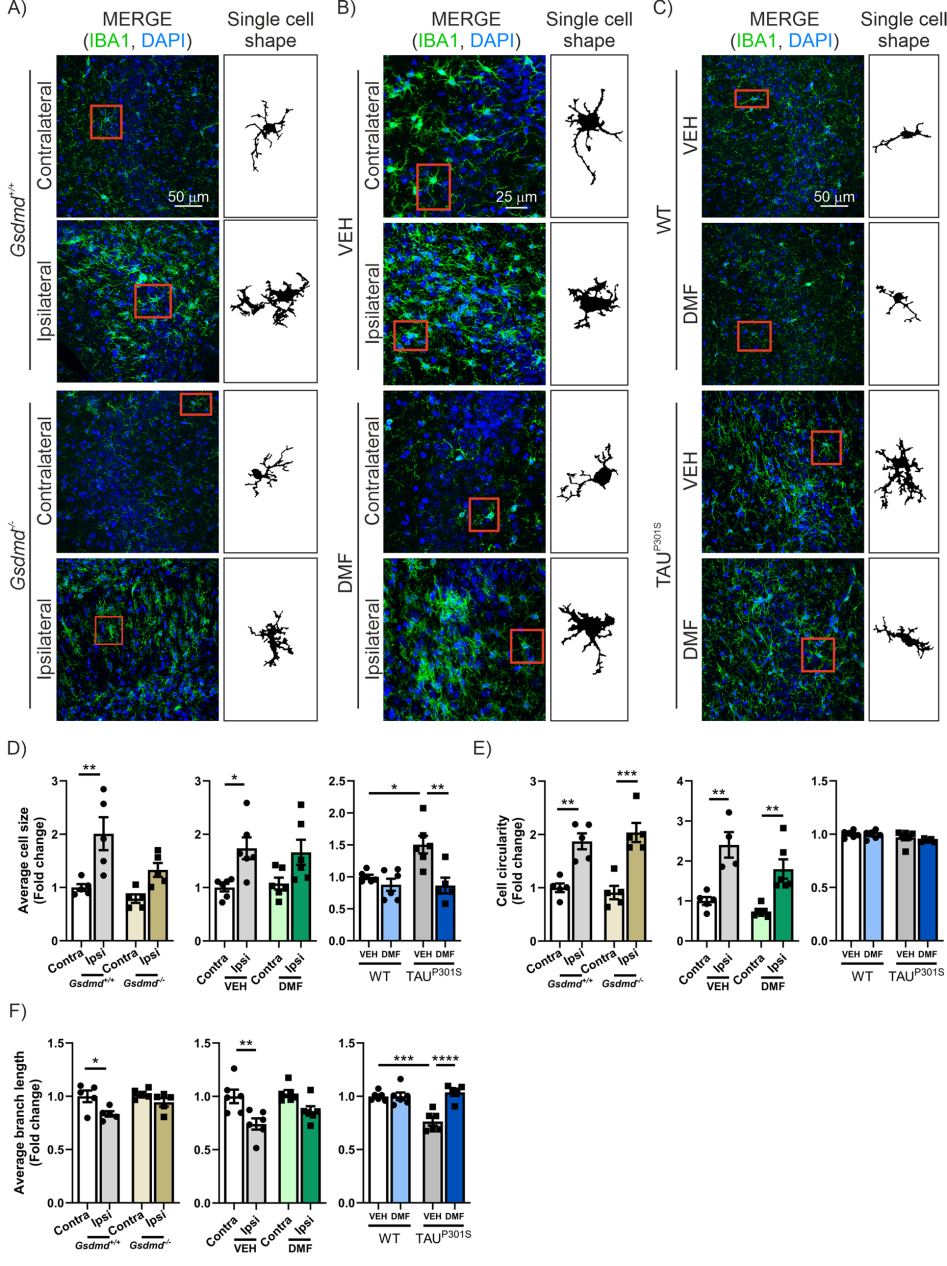
GSDMD deficiency slightly reduces reactive microglial cell size and DMF treatment mitigates microglial activation in both tauopathy mouse models. Immunostaining of IBA1 in the CA3 region of (**A**) TAU^P301L^ overexpressing GSDMD-deficient mice, (**B**) AAV-TAU^P301L^ mice treated with VEH or DMF and (**C**) Tg-TAU^P301S^ mice treated with VEH or DMF. On the right side of each image, the shape of a selected sample cell (red square) is represented. Quantification of (**D**) the average cell size, (**E**) the mean circularity, (**F**) the average branch length of microglial cells of TAU^P301L^ overexpressing GSDMD-deficient mice (yellow tones), AAV-TAU^P301L^ mice treated with VEH or DMF (green tones) and Tg-TAU^P301^S mice treated with VEH or DMF (blue tones). Bars represent the mean of 5–6 samples ± SEM. Asterisks indicate significant differences of *p < 0.05; **p < 0.01; ***p < 0.001; ****p < 0.0001 comparing each group by two-factor ANOVA test followed by Bonferroni post-test

Following this, we analyzed the impact of GSDMD deficiency on the neuroinflammatory process associated with TAU, focusing on astrogliosis, microgliosis, and other pro-inflammatory markers. Our results indicate that GSDMD deficiency has a minimal effect on the number of GFAP^+^ cells or the mRNA expression levels of *Gfap* induced by TAU overexpression (Supplementary Figure S2C, D). Regarding microgliosis, the absence of GSDMD slightly reduces the number of IBA1^+^ cells (without reaching statistical significance) but does lead to a statistically significant decrease in *Iba1* mRNA expression levels (Supplementary Figure S2C-E). Additionally, we performed an analysis of microglia morphology, as changes in their structure are related to their functionality (Fig. 6A) [73, 74]. For example, reactive microglia retract their branches and adopt a less complex structure with an enlarged cell body, a morphology associated with increased phagocytosis. It has been described that retraction of microglial branches increases the efficiency of migration to the site of damage, and increases phagocytic abilities. In this sense, we demonstrated that TAU overexpression significantly increases the average size of microglia in WT animals while a modest effect was observed in GSDMD deficient mice (Fig. 6D). Moreover, we observed that TAU overexpression strongly increases the circularity of microglia (Fig. 6E) in WT mice. Our results reveal that GSDMD deficiency does not lead to significant changes in circularity, but normalizes the length of the branches (Fig. 6F). These results suggest that GSDMD deficiency promotes a restoration of normal microglia morphology in tauopathy. Additionally, we observed a highly significant reduction in the levels of the pro-inflammatory cytokines *Il6* and *Cxcl5* (Supplementary Figure S2F and G, respectively). In the case of *Tnfa*, *Cxcr3*, and *Olr1* expression levels, we also detected a decrease, although it did not reach statistical significance (Supplementary Figure S2H-J, respectively).

### GSDMD absence reduces the expression of synaptic plasticity markers in the CA3/hippocampus, which is exacerbated by TAU overexpression

**Fig. 7:**
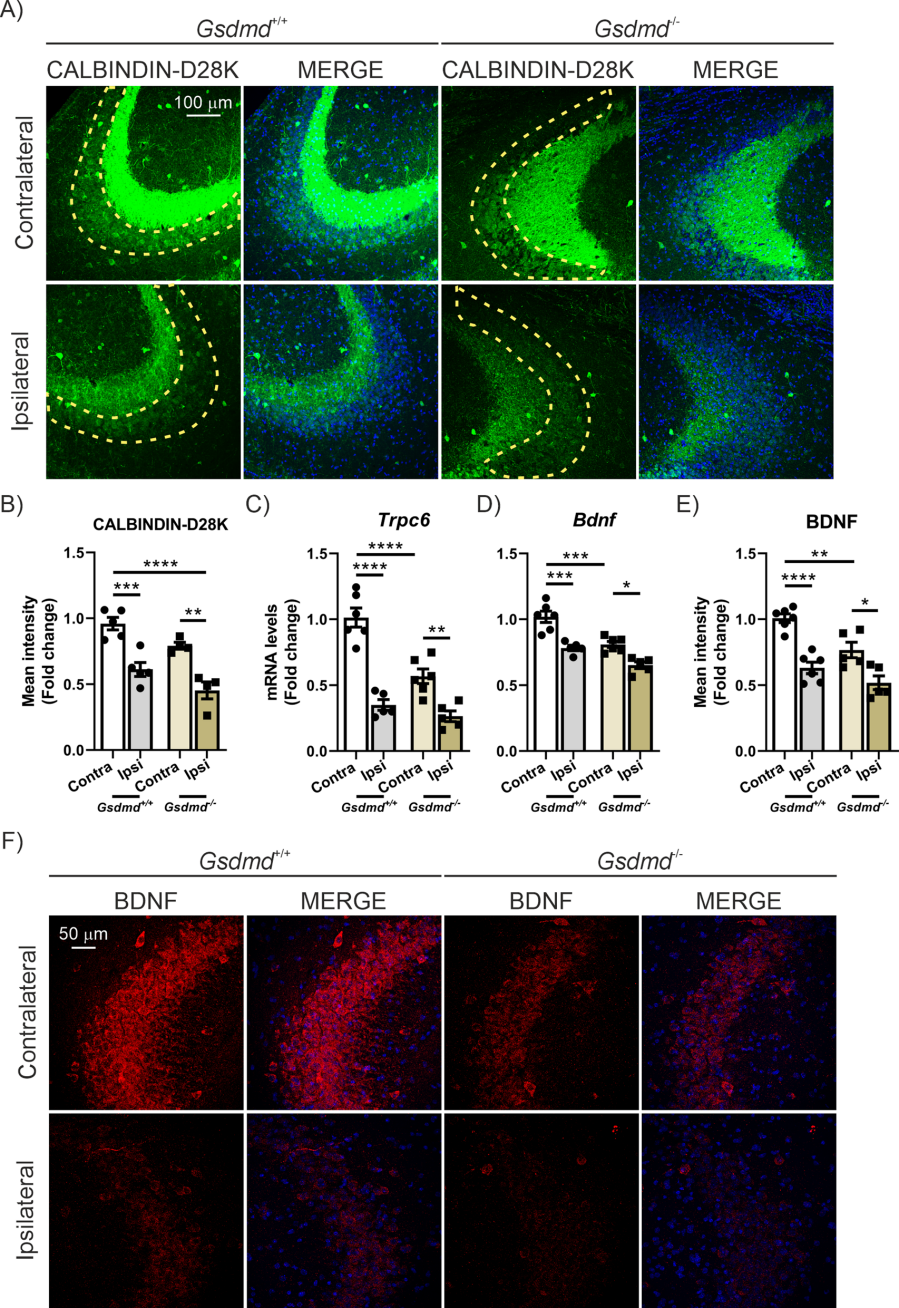
GSDMD deficient mice show decreased expression of synaptic plasticity markers regardless of TAU overexpression. **A** Immunostaining of CALBINDIN-D28K in the CA3 region of *Gsdmd*^+*/*+^ or *Gsdmd*^*−/−*^ mice that overexpress TAU^P301L^. **B** Quantification of CALBINDIN-D28K fluorescence intensity. Analysis of (**C**) *Trcp6* and (**D**) *Bdnf* mRNA levels in hippocampus. **E** Quantification of BDNF fluorescence intensity. **F** Immunostaining of BDNF in the CA3 region. Bars represent the mean of 4–5 samples ± SEM. Asterisks indicate significant differences of *p < 0.05; **p < 0.01; ***p < 0.001; ****p < 0.0001 comparing each group by two-factor ANOVA test followed by Bonferroni post-test

Considering that the absence of GSDMD had a beneficial effect on pyroptosis and the neuroinflammatory process, we next investigated its impact on synaptic plasticity. To our surprise, we observed that GSDMD deficiency in control conditions caused a reduction in CALBINDIN-D28K expression (which did not reach statistical significance) (Fig. 7A, B) and a highly significant decrease in the mRNA expression levels of *Trpc6* (44%) and *Bdnf* (20.7%) (Fig. 7C, D), compared to WT animals. In the case of BDNF, these results were further confirmed at the protein level through immunofluorescence analysis (Fig. 7E, F). The overexpression of TAU significantly reduces CALBINDIN-D28K expression levels by 36.3%, whereas in the absence of GSDMD, this reduction reaches 42.7% (Fig. 7A, B). Regarding *Trpc6* mRNA levels, TAU overexpression decreases expression by 65.3%, while in the absence of GSDMD, the reduction is 53.3% (Fig. 7C). Similarly, *Bdnf* mRNA expression levels are significantly reduced by 23.2% due to TAU overexpression, and in the absence of GSDMD, this reduction is 19.5% (Fig. 7D), with similar results observed in the IF analysis (Fig. 7E, F). This means that *Trpc6* and *Bdnf* levels are already decreased in the absence of GSDMD, and that TAU overexpression does not cause such a drastic reduction in the absence of GSDMD as it does in WT mice. All these changes occur without alterations in the number of cells in the CA3 area (Supplementary Figure S3A) or changes in TAU phosphorylation levels (Supplementary Figure S4A–B). These data suggest that although GSDMD deficiency is beneficial in reducing TAU-induced pro-inflammatory markers, this protein is necessary for proper synaptic plasticity function.

### Preventive treatment with DMF reduces pyroptosis/neuroinflammation induced by TAU overexpression and correlates with improvement in synaptic plasticity in the AAV-TAU^P301L^ model

It has been reported several posttranslational modifications that are crucial in regulating GSDMD pore-forming capacity, such as succination, palmitoylation, and oxidation, target specific cysteine residues. Indeed, several GSDMD inhibitors that affect its cysteines have been identified to date, including disulfiram, necrosulfonamide (NSA), and dimethyl fumarate (DMF) [75]. In the cases of disulfiram and necrosulfonamide, it has been reported that, as cysteine-reactive compounds, they may have off-target effects. They do not completely block the formation of processed GSDMD, but only the pore function [76, 77]. DMF or the accumulation of endogenous fumarate irreversibly modifies GSDMD at C191 (human) and C192 (mouse) and other cysteine residues, leading to the formation of 2-(succinyl)-cysteine [38]. The succination of GSDMD disrupts its interaction with caspases, inhibits its processing and oligomerization, and ultimately prevents the formation of GSDMD pores and the occurrence of pyroptosis. Interestingly, DMF is also an activator of the NRF2 signaling pathway, and our research group has previously described that this compound can modulate NRF2 through both KEAP1-dependent and KEAP1-independent mechanisms (by regulating glycogen synthase kinase 3β (GSK-3β), as well as its role in modulating TAU-associated pathology [39]. Therefore, we first analyzed the status of the NRF2 pathway in the AAV-TAU^P301L^ and the Tg-TAU^P301S^ (at 8 and 10 months of age) models. We observed that the mRNA levels of *Nfe2l2* and the NRF2-dependent genes *Hmox1, Nqo1, Gpx1,* and *Txn1* were significantly induced by TAU overexpression in the two models (Supplementary Figure S5). In 10-month-old Tg-TAU^P301S^ mice, which exhibit a more severe pathological condition, the NRF2 pathway was more strongly induced compared to 8-month-old counterparts (Supplementary Figure S5B, C). These data indicate that TAU overexpression activates the NRF2 signaling pathway, but this activation is not sufficient to counteract the pathology. Additionally, we analyzed the NRF2 signaling pathway in the hippocampus of GSDMD-deficient mice. The absence of GSDMD has little impact on the expression of NRF2-dependent genes (*Hmox-1*, *Nqo1*, *Gpx1*, and *Txn1*), and no significant differences are observed in TAU-dependent NRF2 pathway induction between the two genotypes (Supplementary Figure S6A–D).

**Fig. 8:**
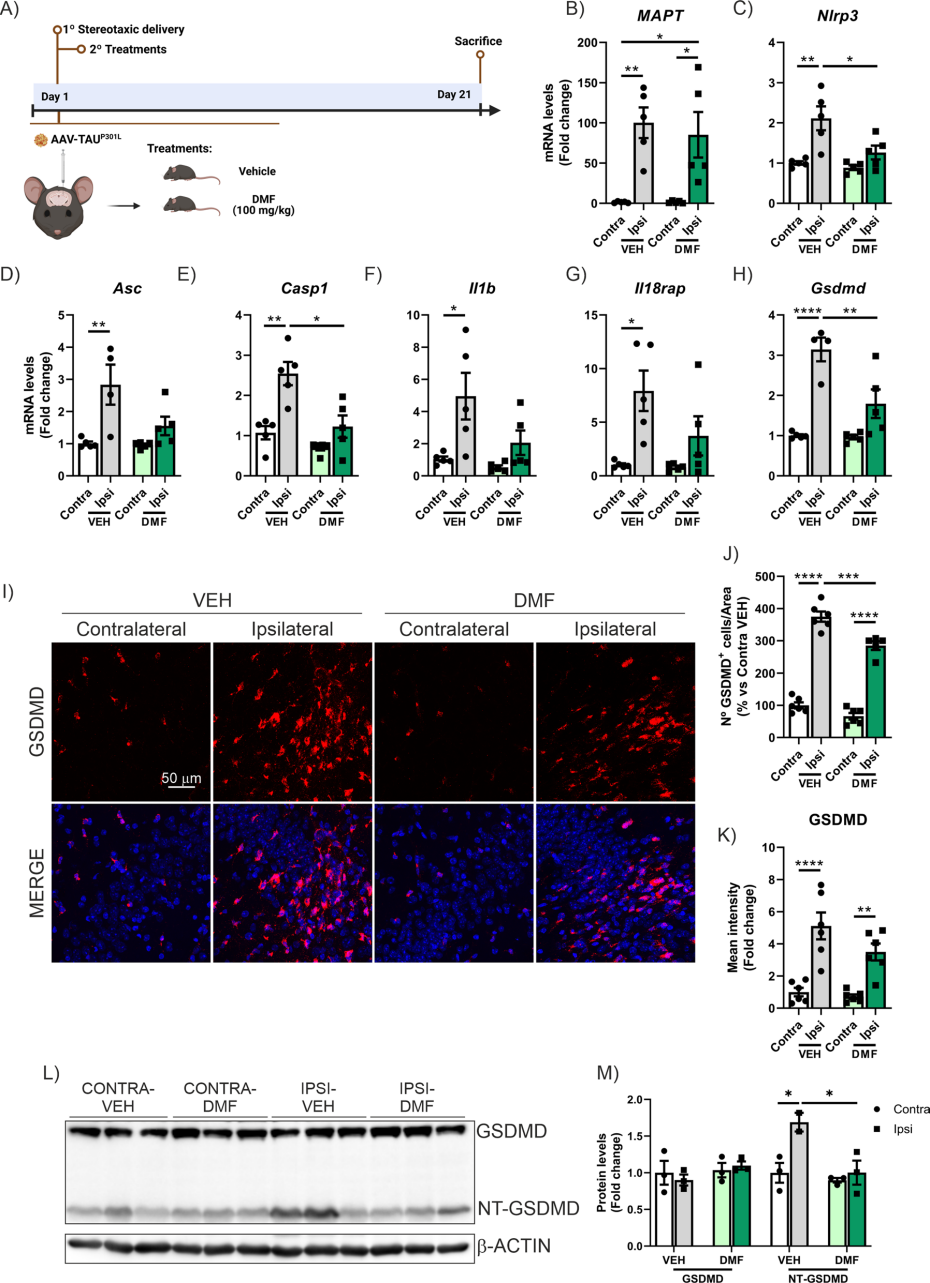
DMF treatment reduces the expression of pyroptosis markers in the AAV-TAU^P301L^ mouse model. **A** Timeline representation of the experimental design: 6 months-old C57BL/6 WT mice were stereotaxic injected with AAV-TAU^P301L^ in the right hippocampus (ipsilateral side) and, at the same day, we started to treat daily with vehicle (VEH) or DMF (100 mg/kg i.g.) for 21 days. Analysis of mRNA levels of (**B**) *MAPT* and (**C**–**H**) genes involved in the pyroptosis process in the hippocampus of AAV-TAU^P301L^ mice treated with VEH or DMF. **I** Quantification of the number of GSDMD^+^ cells. **J** Quantification of GSDMD fluorescence intensity. **K** Immunostaining of GSDMD in the CA3 region. **L**, **M** Protein levels of GDMD and cleavage NT-GSDMD were analyzed by immunoblotting and their respective protein quantifications in hippocampal samples. Bars represent the mean of 5–6 samples ± SEM for mRNA and n = 3 for protein levels. Asterisks indicate significant differences of *p < 0.05; **p < 0.01; ***p < 0.001; ****p < 0.0001 comparing each group by two-factor ANOVA test followed by Bonferroni post-test

To enhance the neuroprotective effect of NRF2 activation, we administered a daily treatment with DMF (100 mg/kg, i.g.) for 3 weeks, starting on the same day the mice were injected with AAV-TAU^P301L^ (see experimental design in Fig. 8A). DMF treatment did not alter *MAPT* expression levels, as similar expression levels were observed between the two experimental groups (Fig. 8B). Next, we analyzed the mRNA expression levels of genes involved in the pyroptosis process. Our results showed that DMF treatment significantly reduced the expression levels of *Nlrp3*, *Casp1*, and *Gsdmd* (Fig. 8C, E and H), while the reduction was moderately decreased and not statistically significant for *Asc*, *Il1b*, and *Il18rap* (Figs. 8D, F and G). Again, we did not observe any changes of *Gsdme* mRNA levels (Supplementary Figure S7B). Then, to define comprehensively the role of NRF2 in the transcriptional regulation of pyroptosis-related genes, we searched for putative ARE sequences in the ChIP-Atlas database, an integrative database covering almost all public data archived in the Sequence Read Archive of NCBI, EBI, and DDBJ, using ChIP-seq data [61] of the mouse (Table 1) genome for *Nlrp3*, *Asc*, *Casp1*, *Il1β*, *Il18rap*, *Aim2*, and *Gsdmd*. The ChIP-Atlas database includes experimental data from chromatin immunoprecipitation (ChIP) analysis of the ARE-binding transcription factors MAFG, MAFF, MAFK, BACH1, and NFE2L2. We used a Python-based bioinformatic analysis to scan this binding region for the consensus ARE, as established in the JASPAR database [59, 78]. Depending on the gene, we detected zero, one, or several putative ARE sequences with a relative score higher than 80%, a commonly used threshold for transcription factor binding-site analysis [79, 80]. These putative ARE sequences in the promotor region have a high degree of similarity with the consensus ARE sequence (N**TGAC**NNN**GCN**) described by [81]. As shown in Table 1, bioinformatic analyses suggested that there are ARE sequences in *Nlrp3*, *Casp1*, *Il1b*, *Il18rap*, and *Aim2*. It is interesting to highlight that it had already been previously demonstrated that NRF2 activation directly suppresses IL-1β synthesis, as NRF2 opposes the transcriptional upregulation of proinflammatory cytokine genes such as *Il1b* and *Il6* [82–84]. In this case, our data suggest that this also may be the case for the genes involved in the pyroptosis process, such as *Nlrp3*, *Casp1*, *Il1b*, *Il18rap*, and *Aim2* but not in the case of *Gsdmd*. To further characterize this regulation, we looked for already described negative regulatory elements adjacent to these putative ARE sequences [85], but none could be found, hinting at another mode of regulation (data not shown).

Regarding the expression levels of the GSDMD protein, we again observe that TAU overexpression increases both the number of GSDMD^+^ cells and their staining intensity, and DMF treatment is able to partially reverse this effect (Fig. 8I–K). These data were corroborated by WB analysis of total hippocampal lysates, confirming that TAU overexpression induces an increase in NT-GSDMD levels, but not in total GSDMD levels, and that treatment with DMF is able to reverse this effect (Fig. 8L-M). The difference in the results obtained by IF and WB is due to the fact that the antibody detects both GSDMD and cleaved NT-GSDMD, and these forms can be clearly distinguished only by WB, not by IF. Although DMF treatment blocks cleavage, GSDMD remains present in the cell, and therefore the effect is not as robust by IF. We also analyzed the processes of astrogliosis and microgliosis, as well as the expression of other pro-inflammatory markers. DMF treatment significantly reduced astrogliosis (both the number of GFAP^+^ cells and *Gfap* mRNA expression levels, Supplementary Figure S7C, D) and microgliosis (*Iba1* mRNA expression levels, Supplementary Figure S7C, E), as well as the levels of *Tnfa*, *Il6*, and *Cxcr3* (Supplementary Figure S7F-H). In the case of *Cxcl5* and *Olr1*, the reduction was not statistically significant (Supplementary Figure S7I-J). We also analyzed the morphology of microglia, where we corroborated that TAU overexpression increased cell size (Fig. 6B, D), its circularity (Fig. 6E), as well as a reduction in branch length (Fig. 6F). And although DMF treatment did not have a significant impact, we did observe a slight decrease in circularity, and a normalization in the length of these branches. Overall, these results suggest that DMF treatment, through a combined effect of NRF2 induction and GSDMD inhibition, exerts a strong anti-pyroptotic and anti-inflammatory effect.

**Fig. 9:**
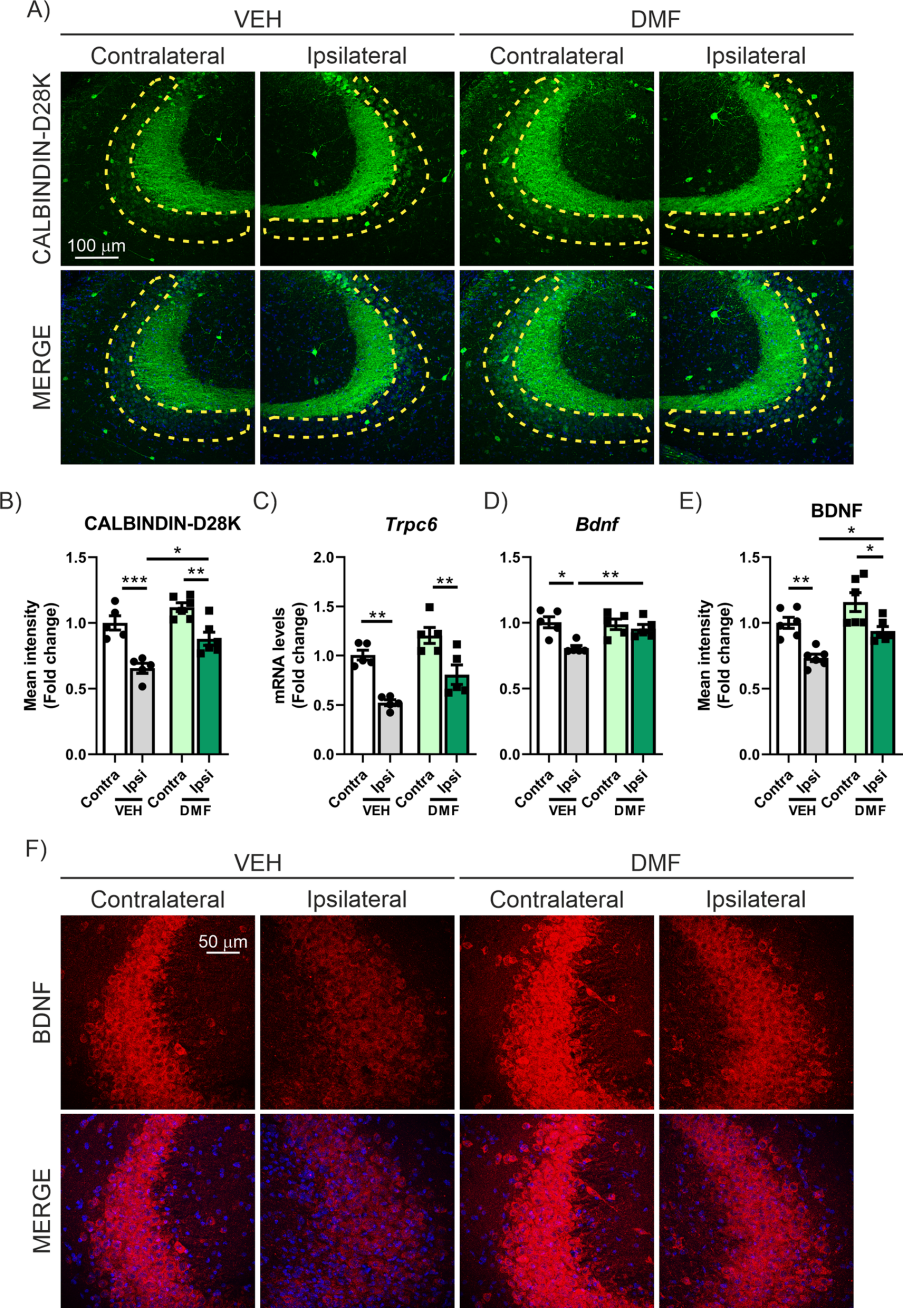
The treatment with DMF prevents the loss of synaptic plasticity markers in the AAV-TAU^P301L^ mouse model. **A** Immunostaining of CALBINDIN-D28K in the CA3 region of control (contralateral) and TAU-overexpressing (Ipsilateral) sides of AAV-TAU^P301L^ mice treated with VEH or DMF. **B** Quantification of CALBINDIN-D28K fluorescence intensity. Analysis of (**C**) *Trcp6* and (**D**) *Bdnf* mRNA levels in hippocampus. **E** Quantification of BDNF fluorescence intensity. **F** Immunostaining of BDNF in the CA3 region. Bars represent the mean of 4–5 samples ± SEM. Asterisks indicate significant differences of *p < 0.05; **p < 0.01; ***p < 0.001 comparing each group by two-factor ANOVA test followed by Bonferroni post-test

Regarding synaptic plasticity, we observed that DMF treatment was able to partially reverse the loss of CALBINDIN-D28K expression in the CA3 region of the hippocampus (Fig. 9A, B). In the case of *Trpc6* mRNA expression levels, the treatment did not produce a statistically significant change, and only a trend was observed: while TAU overexpression led to a 48% reduction in VEH-treated mice, DMF treatment mitigated this loss to 19.7% (Fig. 9C). Finally, the analysis of BDNF, both at the mRNA and protein levels, indicated that DMF treatment was capable of partially reversing the loss induced by TAU overexpression (Fig. 9D-F). All these changes occur without alterations in the number of cells in the CA3 area (Supplementary Figure S3B). The analysis of phospho-TAU levels corroborated the results previously obtained by Lastres-Becker et al. [52], where we observed that TAU overexpression increases phospho-TAU levels, and treatment with DMF produced a highly significant reduction of these levels (Supplementary Figure S4C–D). In conclusion, our results suggest that DMF treatment can prevent the loss of synaptic plasticity associated with TAU overexpression.

### Neuroprotective and neuromodulatory effect of DMF treatment in Tg-TAU^P301S^ mice

**Fig. 10:**
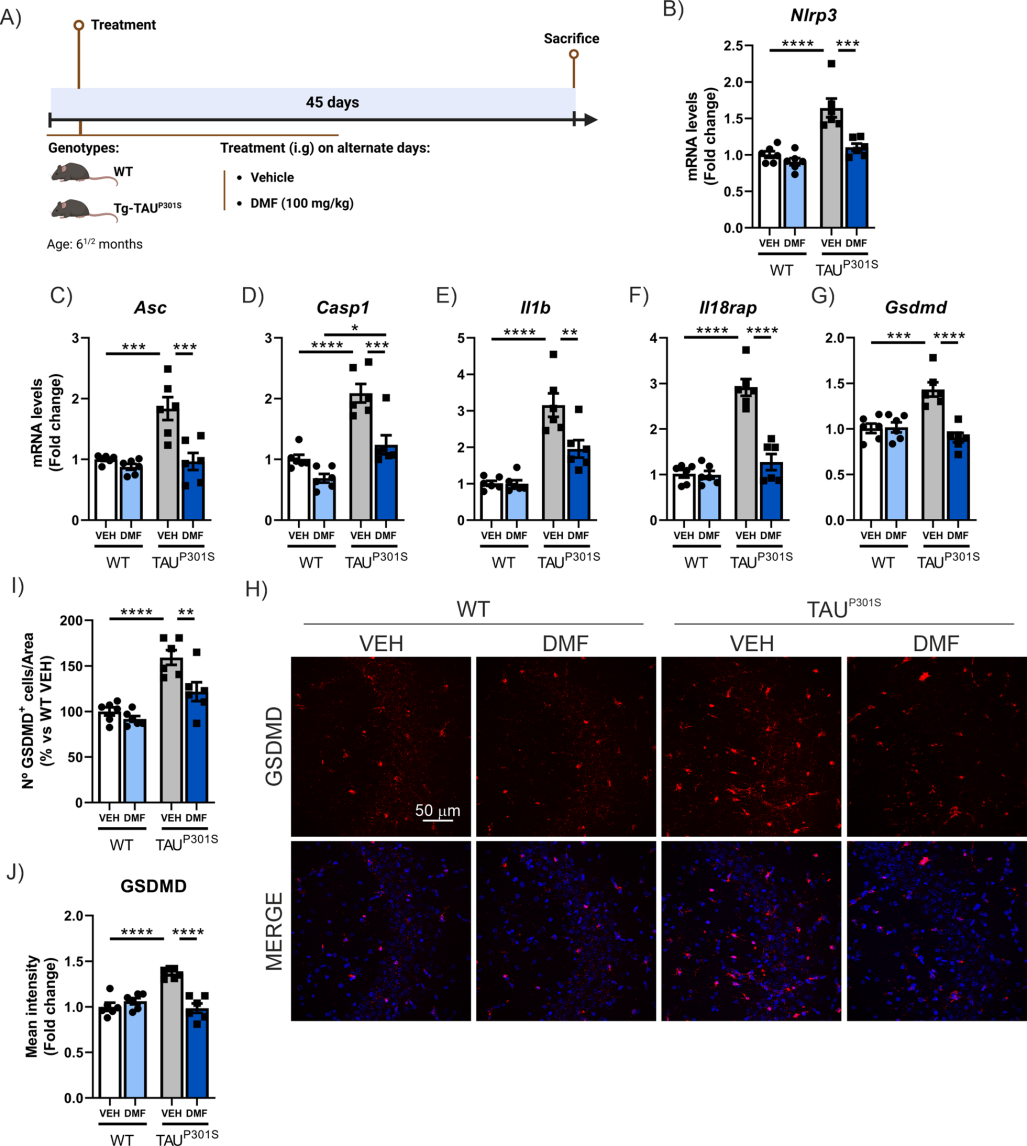
The induction of pyroptosis markers is decreased with DMF treatment in the Tg-TAU^P301S^ mouse model. **A** Timeline representation of the experimental design: 6.5 months-old Tg-TAU^P301S^ mice were treated with DMF (100 mg/kg i.g.) for 45 days in alternate days. **B**–**G** Analysis of mRNA levels of genes involved in the pyroptosis process in the hippocampus of 8-month-old Tg-TAU^P301S^ mice and wild-type mice treated with VEH or DMF. **H** Quantification of the number of GSDMD^+^ cells. **I** Quantification of the fluorescence intensity of GSDMD. **J** Immunostaining of GSDMD in the CA3 region. Bars represent the mean of 5–6 samples ± SEM. Asterisks indicate significant differences of *p < 0.05; **p < 0.01; ***p < 0.001; ****p < 0.0001 comparing each group by two-factor ANOVA test followed by Bonferroni post-test

Finally, we wanted to analyze whether DMF treatment could not only prevent neurodegeneration but also have a neuroprotective effect in a model where the neurodegenerative process was already established. To this end, we treated 6.5-month-old Tg-TAU^P301S^ mice with DMF (100 mg/kg i.g.) on alternating days for 45 days (until they reached 8 months of age) (see experimental design in Fig. 10A). First, we analyzed the NRF2 signaling pathway in the hippocampus of DMF treated mice. Since the samples were collected 16 h after the last administration, we observe only a slight, non-statistically significant induction of NRF2-dependent enzymes with delayed kinetics, such as *Nqo1* and *Gpx1*, although there is a trend suggesting that DMF treatment may activate the NRF2 pathway (Supplementary Figure S6E–H). In WT mice, DMF treatment has minimal effect on the mRNA expression of genes involved in the pyroptosis process (*Nlrp3*, *Asc*, *Casp1*, *Il1b*, *Il18rap*, and *Gsdmd*) (Fig. 10B-G). However, in Tg-TAU^P301S^ mice, where pyroptosis is induced, DMF treatment is able to reverse this effect almost to WT levels. However, TAU overexpression in these mice causes no changes in *Aim2* (Supplementary Figure S8A) or *Gsdme* levels (Supplementary Figure S8B). A more detailed analysis, revealed that the number of GSDMD^+^ cells and the intensity of GSDMD staining is reversed by DMF treatment (Fig. 10H-J). The impact of DMF treatment on the broader neuroinflammatory process is also highly significant. Astrogliosis and microgliosis, both assessed by mRNA levels and the number of positive cells, induced by TAU overexpression are significantly reduced by DMF treatment (Supplementary Figure S8C-E). We also analyzed the morphology of microglia, observing that in this model, TAU^P301S^ overexpression induced an increase in cell size (Fig. 6C, D), but a significant decrease in their length (Fig. 6F). All these alterations in microglia morphology were reversed by DMF treatment. In this case, we did not observe changes in microglia circularity associated with either TAU or DMF treatment (Fig. 6E). Additionally, it is noteworthy that DMF treatment normalizes the mRNA expression of pro-inflammatory genes such as *Tnfa*, *Il6*, *Cxcr3*, and *Cxcl5* (Supplementary Figure S8F-I). In this model, no significant changes were observed in *Olr1* expression (Supplementary Figure S8J). Altogether, these results suggest that DMF treatment may reverse the neuroinflammatory state induced by TAU overexpression.

**Fig. 11:**
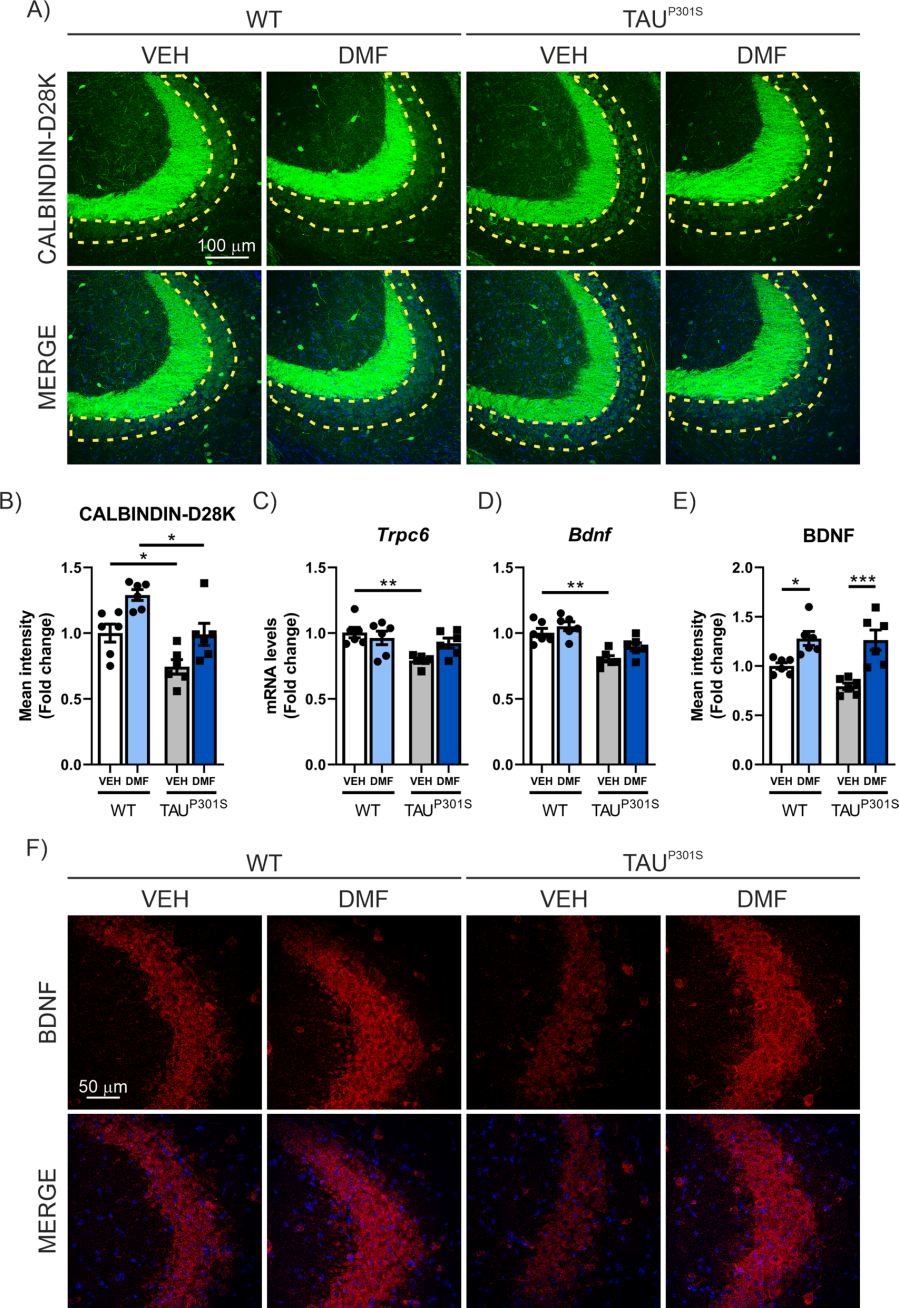
The treatment with DMF in the Tg-TAU^P301S^ mouse model restores the loss of synaptic plasticity markers. **A** Immunostaining of CALBINDIN-D28K in the CA3 region in 8-month-old Tg-TAU^P301S^ mice and wild-type mice treated with VEH or DMF. **B** Quantification of CALBINDIN-D28K fluorescence intensity. Analysis of (**C**) *Trcp6* and (**D**) *Bdnf* mRNA levels in hippocampus. **E** Quantification of BDNF fluorescence intensity. **F** Immunostaining of BDNF in the CA3 region. Bars represent the mean of 5–6 samples ± SEM. Asterisks indicate significant differences of *p < 0.05; **p < 0.01; ***p < 0.001 comparing each group by two-factor ANOVA test followed by Bonferroni post-test

Finally, we analyzed the impact of modulating the neuroinflammatory process with DMF on the loss of neuronal plasticity markers associated with TAU overexpression. As shown in Fig. 11A, B, the loss of CALBINDIN-D28K expression (25.7%) is restored to WT levels by DMF treatment. In the case of mRNA expression levels for *Trpc6*, DMF treatment reverses the loss of expression induced by TAU overexpression from 21.23% to only 7.9% (Fig. 11C). Similar results were observed in the analysis of mRNA levels for *Bdnf*, where DMF treatment reverses the loss from 20% to only 10.8% (Fig. 11D). At the protein level, the loss of BDNF expression induced by TAU overexpression was fully reversed by DMF treatment (Fig. 11E-F). All these changes occur without alterations in the number of cells in the CA3 area (Supplementary Figure S3C). The analysis of phospho-TAU levels where similar as for the AAV-TAU^P301L^ model, where we observed that Tg-TAU^P301S^ showed increased phospho-TAU levels, and treatment with DMF produced a highly significant reduction of these levels (Supplementary Figure S4E–F). The results obtained demonstrate that DMF treatment can have a protective effect both at the inflammatory and synaptic plasticity levels against TAU overexpression.

## Discussion

Although the contribution of TAU to the process of degeneration and neuroinflammation is well-documented [19, 20, 22, 70, 86, 87], its involvement in pyroptosis remains controversial. In this study, we demonstrate for the first time a significant increase in the expression levels of genes involved in the pyroptosis process in the hippocampus of AD patients. We also observed a substantial increase in inflammasome/pyroptosis activation induced by TAU in both an AAV-TAU^P301L^ model and Tg-TAU^P301S^ (PS19) mice, where this effect was exacerbated over time due to progressive degeneration. Analyzing the role of GSDMD in TAU-associated neuroinflammation highlights its importance, as GSDMD deletion significantly reduces TAU-induced neuroinflammation. However, GSDMD deficiency has a downside: a decrease in synaptic plasticity in the hippocampus. This underscores the significance of pharmacological inhibition of GSDMD, particularly through treatment with DMF. Interestingly, although DMF also targets the cysteine residues of GSDME, this GSDM does not appear to be altered in these disease models, suggesting that the effect of DMF is primarily focused on GSDMD and NRF2. Our findings reveal that DMF treatment is capable of both preventing and reversing the TAU-induced neurodegenerative and neuroinflammatory processes. This study highlights the potential of pyroptosis modulation as a novel therapeutic strategy for tauopathies.

Although much has been written about the involvement of the pyroptosis process in AD, to date, studies linking pyroptosis to AD have primarily been conducted on cerebrospinal fluid (CSF) samples, where a significant increase in GSDMD levels has been reported, suggesting its potential as a biomarker for AD [88]. Another study has also observed the activation of the NLRP3 and NLRP1 inflammasomes in monocytes from AD patients stimulated with LPS and Aβ42 [21]. Additionally, increased caspase-1 activation has been detected in frontal cortex and hippocampus samples from AD patients, as shown by Western blot analysis at the protein level [22]. So far, only one study has analyzed four microarray datasets in the hippocampus of AD patients from the GEO database [34]. Therefore, the analysis performed by our group on the genes involved in the pyroptosis process directly in hippocampal samples from AD patients is highly significant, as it provides direct evidence of a correlation between the pathology and this type of cell death.

One question that arises regarding the activation of the pyroptosis process in AD is what induces it. Previous studies suggest that TAU protein is responsible for activating the NLRP3 inflammasome, both in mouse models of tauopathy and in samples from patients with frontotemporal dementia (FTD) caused by TAU alterations [19]. These findings led us to analyze the pyroptosis process in two mouse models of tauopathy (AAV-TAU^P301L^ and Tg-TAU^P301S^). Our analysis not only examines the inflammasome activation but also the pyroptosis pathway, providing a more comprehensive picture of the TAU-induced process.

Previously, it had been described that aggregated TAU was capable of activating the NLRP3 inflammasome, exacerbating seeded TAU pathology, and that this process was inhibited by MCC950, an NLRP3 inhibitor [69]. Subsequent studies highlighted the importance of the NLRP3 inflammasome in tauopathy, as the absence of NLRP3 improved the neurodegenerative phenotype in Tg-TAU^P301S^ mice, as well as TAU propagation and hippocampal atrophy [17]. Similarly, NLRP3 deficiency in the Tg-TAU^P301S^ model promoted pericyte survival and improved cerebrovascular function [16]. However, another similar study yielded opposite results, suggesting that NLRP3 inflammasome activation was dispensable for TAU pathology [89]. It is important to remark that in this last study, neither neuroinflammation nor neurodegeneration/synaptic plasticity was analyzed. Instead, only phospho-TAU levels were determined by immunohistochemistry using AT100 and AT8 in the brainstem, midbrain, cortex, and spinal cord of double-transgenic mice. Our study provides a comprehensive analysis of the entire neuroinflammatory profile, as well as pyroptosis, in two different tauopathy models, emphasizing the key role of pyroptosis in the TAU-induced degenerative process.

Currently, there is limited information on the role of GSDMD in the brain or microglia. It has been reported that GSDMD knockdown attenuates the migration and phagocytic activity of microglia [90]. Considering that one of the main functions of microglia is synaptic pruning, our results suggest that GSDMD deficiency leads to impaired microglial activity, affecting synaptic plasticity (decreased levels of CALBINDIN-D28K, TRPC6 and BDNF). These already significantly reduced levels on the contralateral side in knockout mice compared to WT, further support the presence of deficits in the knockout animals independent of TAU. The lifelong global knockout leads to clear maladaptive consequences. But a more exhaustive analysis of this process will be needed in future experiments including the analysis of why GSDMD-deficient mice exhibit behavioral alterations. Besides, it has also been described that GSDMD and caspase-1/11 deficient mice show deficits in brain inflammation [91], and that NLRP3 and microglial GSDMD deficiency markedly attenuate lipopolysaccharide-induced BBB breakdown [92]. These findings are consistent with the results of our study, where we observed that GSDMD deficiency reduces the TAU-induced neuroinflammatory response.

Due to the importance of the pyroptosis process in the TAU-associated neurodegenerative profile, it emerges as a potential pharmacologically targetable therapeutic strategy for tauopathies. Among the inhibitors described to date, DMF holds significant importance, as it is already used in clinical practice for patients with multiple sclerosis [93–97], making its repositioning for tauopathies a feasible option. However, it is important to consider that DMF is not solely a GSDMD inhibitor; it also has multiple actions, including activating the NRF2 transcription factor, enhancing both antioxidant and anti-inflammatory responses. Previously, our group described that DMF has beneficial effects in the AAV-TAU^P301L^ tauopathy model through two mechanisms: DMF induces NRF2 pathway involving KEAP1, as well as PI3K/AKT/GSK-3-dependent pathways [52]. Interestingly, DMF treatment modulates GSK-3β activity, the main kinase involved in TAU phosphorylation. Indeed, we previously observed that DMF treatment reduced phospho-TAU levels in this tauopathy model [52]. These data suggest that DMF treatment may act at three different levels, which are key aspects of the TAU neurodegenerative process: (1) modulating of GSK-3β activity, thereby reducing phospho-TAU levels; (2) enhancing the antioxidant capacity of the NRF2 signaling pathway; (3) inhibiting the inflammatory process on two fronts—by activating NRF2’s anti-inflammatory response and by inhibiting GSDMD in the pyroptosis process. Our study also suggests these processes may be interconnected through NRF2-mediated transcriptional inhibition of pyroptosis genes, although further mechanistic studies are needed to clarify its role. The fact that DMF treatment is able to modulate several mechanisms involved in the pathology should be considered a positive aspect, given the multifactorial nature of the neurodegenerative process. Targeting a single molecule in such a complex process is very unlikely to be effective. For this reason, in recent years the focus has shifted towards multi-target compounds. Importantly, although DMF treatment has side effects, such as gastrointestinal side-effects like abdominal pain and flushing issues [98, 99], or leukopenia [94], considering that patients with tauopathies currently lack any effective treatment, the potential repurposing of DMF for these patients may be worth exploring. It is important to note that the effect of DMF is not more pronounced in the Tag-TAU^P301S^ model; rather, the inflammatory process is more severe in the AAV-TAU^P301L^ model. Therefore, it is easier to reverse neuroinflammation/pyroptosis in the transgenic model than in the AAV-TAU^P301L^ model.

## Conclusions

Our findings strongly support the involvement of pyroptosis in the neurodegenerative process of tauopathies, with increased expression of key pyroptosis-related genes observed in hippocampal samples from AD patients and tauopathy mouse models. TAU overexpression was shown to drive this inflammatory cascade, particularly through microglial activation and GSDMD upregulation, which correlated with reduced synaptic plasticity markers. Moreover, GSDMD deficiency and pharmacological inhibition with DMF demonstrated a partial reduction in pyroptosis and inflammation, highlighting GSDMD’s complex role in balancing inflammation and synaptic function. These results emphasize pyroptosis as a potential therapeutic target in tauopathies.

## Supplementary Information

Additional file 1 (Supplementary Figure 1. Both tauopathy mouse models show an increase of neuroinflammation markers driven by TAU overexpression. Analysis of (A) Aim2 and (B) Gsdme mRNA levels in hippocampal samples from AAV-TAUP301L mice (light green) and in 8-month-old (light blue) and 10-month-old (dark blue) Tg-TAUP301S mice. (C) Immunostaining of GFAP (red) and IBA1 (green) in the CA3 region of both FTD-TAU models. (D) Quantification of the number of GFAP+ cells. (E) Analysis of Gfap mRNA levels. (F) Quantification of the number of IBA1+ cells. (G) Analysis of Iba1 mRNA levels. Bars represent the mean of 5-7 samples ±SEM. Asterisks indicate significant differences of **p<0.01; ***p<0.001; ****p<0.0001 comparing each group by Student's t-test.)

Additional file 2 (Supplementary Figure 2. GSDMD-deficient mice have decreased TAU-induced neuroinflammation markers compared to WT mice. Analysis of mRNA levels of (A) Aim2 and (B) Gsdme in the hippocampus of Gsdmd+/+ and Gsdmd-/- mice that overexpress TAUP301L on the ipsilateral side. (C) Immunostaining of GFAP (red) and IBA1 (green) in the CA3 region. (D) Quantification of the number of GFAP+ cells and Gfap mRNA levels. (E) Quantification of the number of IBA1+ cells and Iba1 mRNA levels. Analysis of the mRNA levels of (F) Il6, (G) Cxcl5, (H) Tnfa, (I) Cxcr3 and (J) Olr1. Bars represent the mean of 4-5 samples ±SEM. Asterisks indicate significant differences of *p<0.05; **p<0.01; ***p<0.001; ****p<0.0001 comparing each group by two-factor ANOVA test followed by Bonferroni post-test.)

Additional file 3 (Supplementary Figure 3. GSDMD deficiency, the overexpression of TAU and the treatment with DMF do not produce changes in the number of cells in CA3 area of hippocampus. Quantification of cells in CA3 region of (A) Gsdmd+/+ and Gsdmd-/- mice that overexpress TAUP301L on the ipsilateral side, (B) AAV-TAUP301L mice treated with VEH or DMF and (C) 8-month-old Tg-TAUP301S mice and WT mice treated with VEH or DMF. Bars represent the mean of 4-6 samples ±SEM.)

Additional file 4 (Supplementary Figure 4. GSDMD deficiency does not alter TAU levels in AAV-TAUP301L mice and DMF treatment reduces TAU phosphorylation in hippocampus of both tauopathy mouse models. Immunostaining of total TAU (red) and p-TAUSer202,Thr205 (green) in the CA3 region of (A) Gsdmd+/+ and Gsdmd-/- mice that overexpress TAUP301L on the ipsilateral side, (C) AAV-TAUP301L mice treated with VEH or DMF and (E) 8-month-old Tg-TAUP301S mice and WT mice treated with VEH or DMF. Quantification of p-TAUSer202,Thr205/total TAU ratio of (B) Gsdmd+/+ and Gsdmd-/- mice that overexpress TAUP301L on the ipsilateral side, (D) AAV-TAUP301L mice treated with VEH or DMF and (F) 8-month-old Tg-TAUP301S mice and WT mice treated with VEH or DMF. Bars represent the mean of 4-6 samples ±SEM. Asterisks indicate significant differences of ***p<0.005; ****p<0.001 comparing each group by t-Student test.)

Additional file 5 (Supplementary Figure 5. The overexpression of TAU induces NRF2 signaling pathway in hippocampus in both tauopathy mouse models. Analysis of NRF2-dependent enzyme mRNA levels in (A) AAV-TAUP301L mice (Contra: control side; Ipsi: TAU-overexpressing side); (B) 8-months-old and (C) 10-months-old transgenic TAUP301S mice. Bars represent the mean of 4-5 samples ±SEM. Asterisks indicate significant differences of *p<0.05; **p<0.01; ***p<0.005; ****p<0.001 comparing each group by t-Student test.)

Additional file 6 (Supplementary Figure 6. NRF2 signaling pathway in hippocampus is not altered by GSDMD deficiency and activated by DMF treatment. Analysis of mRNA levels of (A) Hmox1, (B) Nqo1, (C) Gpx1 and (D) Txn1 in the hippocampus of Gsdmd+/+ and Gsdmd-/- mice that overexpress TAUP301L on the ipsilateral side. Analysis of mRNA levels of (E) Hmox1, (F) Nqo1, (G) Gpx1 and (H) Txn1 in the hippocampus of 8-month-old Tg-TAUP301S mice and WT mice treated with VEH or DMF. Bars represent the mean of 4-5 samples ±SEM. Asterisks indicate significant differences of *p<0.05; **p<0.01; ***p<0.005 comparing each group by two-factor ANOVA test followed by Bonferroni post-test.)

Additional file 7 (Supplementary Figure 7. DMF treatment reduces the neuroinflammation in the hippocampus in the AAV-TAUP301L mouse model. Analysis of the mRNA levels of (A) Aim2 and (B) Gsdme in the hippocampus of AAV-TAUP301L mice treated with VEH or DMF. (C) Immunostaining of GFAP (red) and IBA1 (green) in the CA3 region. (D) Quantification of the number of GFAP+ cells and Gfap mRNA levels. (E) Quantification of the number of IBA1+ cells and Iba1 mRNA levels. Analysis of the mRNA levels of (F) Il6, (G) Tnfa, (H) Cxcr3, (I) Cxcl5 and (J) Olr1. Bars represent the mean of 4-5 samples ±SEM. Asterisks indicate significant differences of *p<0.05; **p<0.01; ***p<0.001; ****p<0.0001 comparing each group by two-factor ANOVA test followed by Bonferroni post-test.)

Additional file 8 (Supplementary Figure 8. The induction of neuroinflammation markers is decreased by the treatment with DMF in the Tg-TAUP301S mouse model. Analysis of (A) Aim2 and (B) Gsdme mRNA levels in the hippocampus of 8-month-old Tg-TAUP301S mice and WT mice treated with VEH or DMF. (C) Immunostaining of GFAP (red) and IBA1 (green) in the CA3 region. (D) Quantification of the number of GFAP+ cells and Gfap mRNA levels. (E) Quantification of the number of IBA1+ cells and Iba1 mRNA levels. Analysis of the mRNA levels of (F) Il6, (G) Tnfa, (H) Cxcr3, (I) Cxcl5 and (J) Olr1. Bars represent the mean of 4-5 samples ±SEM. Asterisks indicate significant differences of *p<0.05; **p<0.01; ***p<0.001; ****p<0.0001 comparing each group by two-factor ANOVA test followed by Bonferroni post-test.)

Additional file 9.

Additional file 10.

Additional file 11.

Additional file 12.

## Data Availability

The data that supports the findings of this study is available by contacting the corresponding author.

## Abbreviations

β-TrCP: β-Transducin repeat-containing E3 ubiquitin-protein ligase.
AAV: Adeno-associated virus.
AD: Alzheimer’s disease.
AIM2: Absent in melanoma 2.
ANOVA: Analysis of variance.
ARE: Antioxidant response element.
ASC: Apoptosis-associated speck-like protein containing a CARD.
BBB: Blood-brain barrier.
BDNF: Brain-derived neurotrophic factor.
Casp1: Caspase 1.
cCREs: Candidate cis-regulatory elements.
ChIP-seq: Chromatin immunoprecipitation sequencing.
CNS: Central nervous system.
*Cxcl5*: Chemokine, CXC motif, ligand 5.
*Cxcr3*: Chemokine, CXC motif, receptor 3.
CUL3/RBX1: Cullin 3 and RING-box protein 1.
DAB: 3,3-Diaminobenzidine.
DAPI: 4′,6-Diamidine-2′-phenylindole.
DMF: Dimethyl fumarate.
DG: Dentate gyrus.
EGFP: Enhanced green fluorescent protein.
FDR: False positive rate.
Fig.: Figure.
FTD: Frontotemporal dementia.
FTLD: Frontotemporal lobar degeneration.
GFAP: Glial fibrillary acidic protein.
*Gpx1*: Glutathione peroxidase 1
GSDMD: GASDERMIN D.
GSDME: GASDERMIN E.
GSK-3β: Glycogen synthase kinase 3β.
*Hmox1*: Heme oxygenase 1.
IBA1: Ionized calcium binding adaptor molecule 1.
IF: Immunofluorescence.
IL-1β: Interleukin 1β.
IL-18: Interleukin 18.
*Il18rap*: Interleukin 18 receptor accessory protein.
*Il6*: Interleukin 6
i.p.: Intraperitoneal.
KEAP1: Kelch-like ECH-associated protein 1.
MAF: Musculo-aponeurotic fibrosarcoma
MAP: Microtubule-associated protein.
*MAPT*: Microtubule-associated protein TAU.
mRNA: Messenger ribonucleic acid.
NLRC4: NLR family, CARD domain-containing 4.
NLRP1: NLR family, pyrin domain-containing 1.
NLRP3: NLR family, pyrin domain-containing 3.
*Nqo1*: NAD(P)H quinone dehydrogenase 1.
NRF2: Nuclear factor erythroid-derived 2-like 2.
NSA: Necrosulfonamide.
LPS: Lipopolysaccharide.
*Olr1*: Oxidized low-density lipoprotein receptor 1.
PFM: Position frequency matrix.
PI3K: Phosphoinositide 3-kinase.
PSP: Progressive supranuclear palsy.
qPCR: Quantitative real time Polymerase Chain Reaction.
RNA: Ribonucleic acid.
ROS: Reactive oxygen species.
SEM: Standard Error of the Mean.
Suppl.: Supplementary.
Tg: Transgenic.
*Tnfa*: Tumor necrosis factor α.
*Txn1*: Thioredoxin 1.
TRPC6: Transient receptor potential canonical 6.
VEH: Vehicle.
WT: Wild type
