# Supplementary figures and images for: GASDERMIN D-mediated pyroptosis as a therapeutic target in TAU-dependent frontotemporal dementia mouse model
Source: J Biomed Sci. 2026 Jan 5;33:6. doi: 10.1186/s12929-025-01210-1 (PMC12766953; doi:10.1186/s12929-025-01210-1)

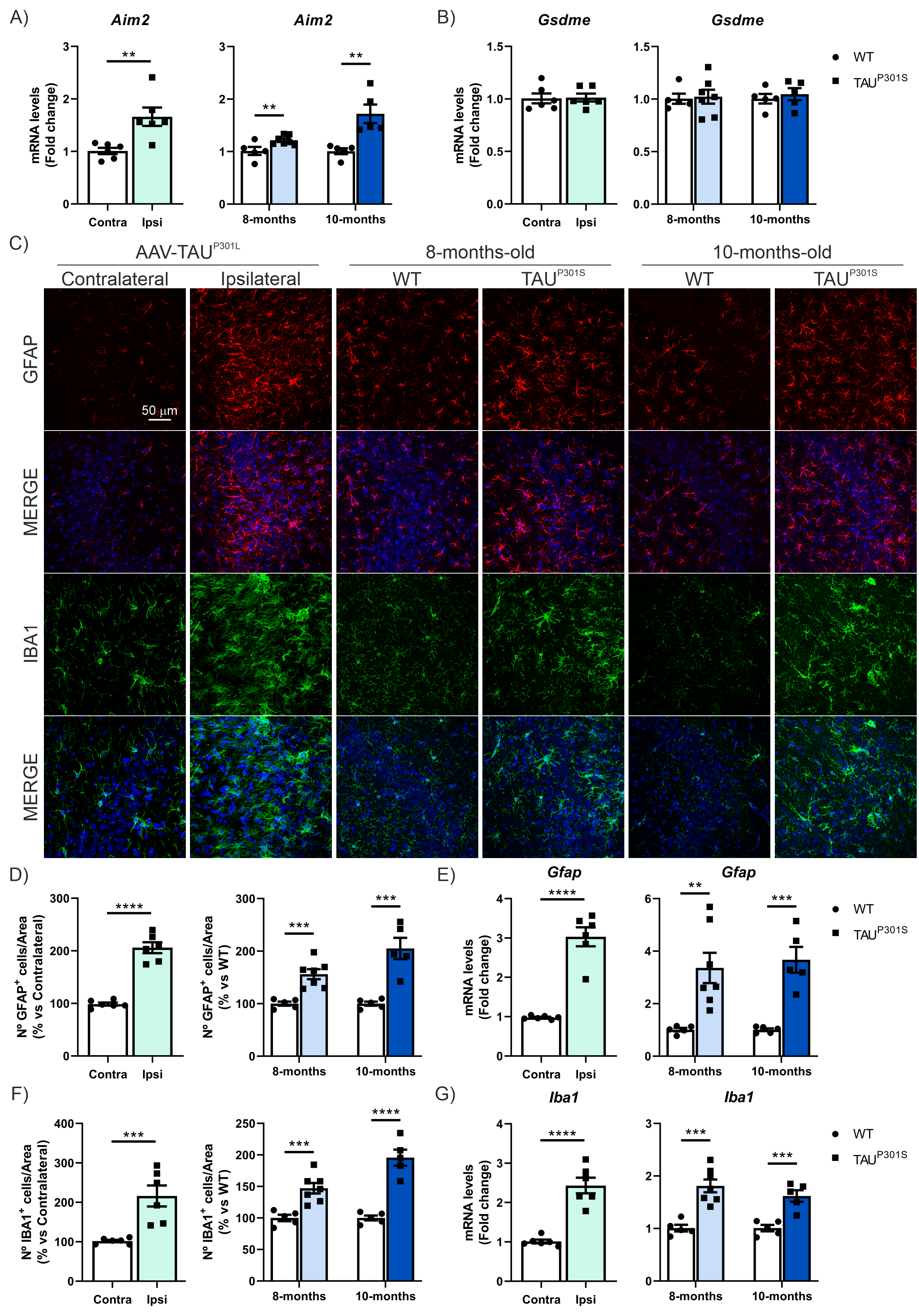

Supplement: Supplementary file 1 — Additional file 1 (Supplementary Figure 1. Both tauopathy mouse models show an increase of neuroinflammation markers driven by TAU overexpression. Analysis of (A) Aim2 and (B) Gsdme mRNA levels in hippocampal samples from AAV-TAUP301L mice (light green) and in 8-month-old (light blue) and 10-month-old (dark blue) Tg-TAUP301S mice. (C) Immunostaining of GFAP (red) and IBA1 (green) in the CA3 region of both FTD-TAU models. (D) Quantification of the number of GFAP+ cells. (E) Analysis of Gfap mRNA levels. (F) Quantification of the number of IBA1+ cells. (G) Analysis of Iba1 mRNA levels. Bars represent the mean of 5-7 samples ±SEM. Asterisks indicate significant differences of **p<0.01; ***p<0.001; ****p<0.0001 comparing each group by Student's t-test.) [file 12929_2025_1210_MOESM1_ESM.tif]

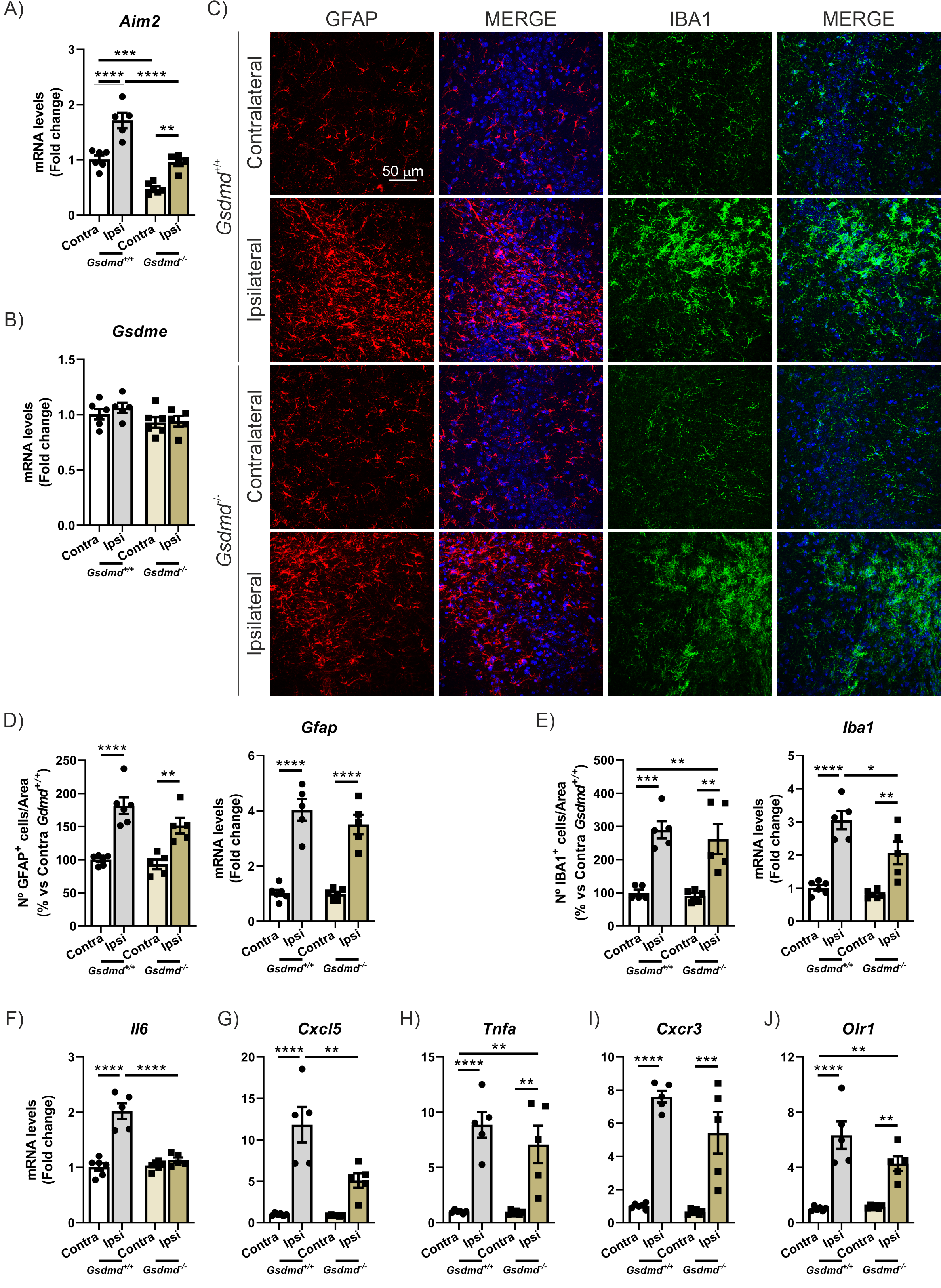

Supplement: Supplementary file 2 — Additional file 2 (Supplementary Figure 2. GSDMD-deficient mice have decreased TAU-induced neuroinflammation markers compared to WT mice. Analysis of mRNA levels of (A) Aim2 and (B) Gsdme in the hippocampus of Gsdmd+/+ and Gsdmd-/- mice that overexpress TAUP301L on the ipsilateral side. (C) Immunostaining of GFAP (red) and IBA1 (green) in the CA3 region. (D) Quantification of the number of GFAP+ cells and Gfap mRNA levels. (E) Quantification of the number of IBA1+ cells and Iba1 mRNA levels. Analysis of the mRNA levels of (F) Il6, (G) Cxcl5, (H) Tnfa, (I) Cxcr3 and (J) Olr1. Bars represent the mean of 4-5 samples ±SEM. Asterisks indicate significant differences of *p<0.05; **p<0.01; ***p<0.001; ****p<0.0001 comparing each group by two-factor ANOVA test followed by Bonferroni post-test.) [file 12929_2025_1210_MOESM2_ESM.tif]

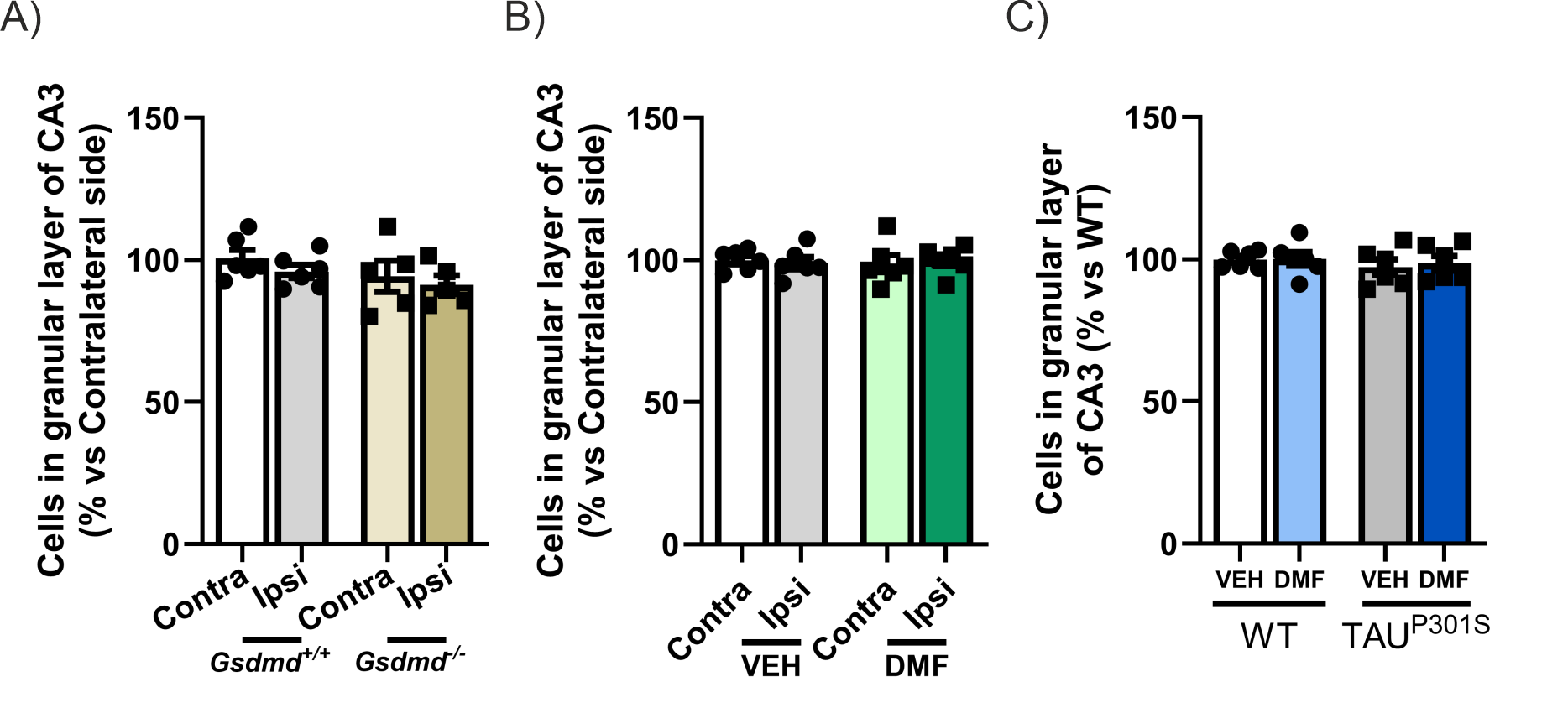

Supplement: Supplementary file 3 — Additional file 3 (Supplementary Figure 3. GSDMD deficiency, the overexpression of TAU and the treatment with DMF do not produce changes in the number of cells in CA3 area of hippocampus. Quantification of cells in CA3 region of (A) Gsdmd+/+ and Gsdmd-/- mice that overexpress TAUP301L on the ipsilateral side, (B) AAV-TAUP301L mice treated with VEH or DMF and (C) 8-month-old Tg-TAUP301S mice and WT mice treated with VEH or DMF. Bars represent the mean of 4-6 samples ±SEM.) [file 12929_2025_1210_MOESM3_ESM.tif]

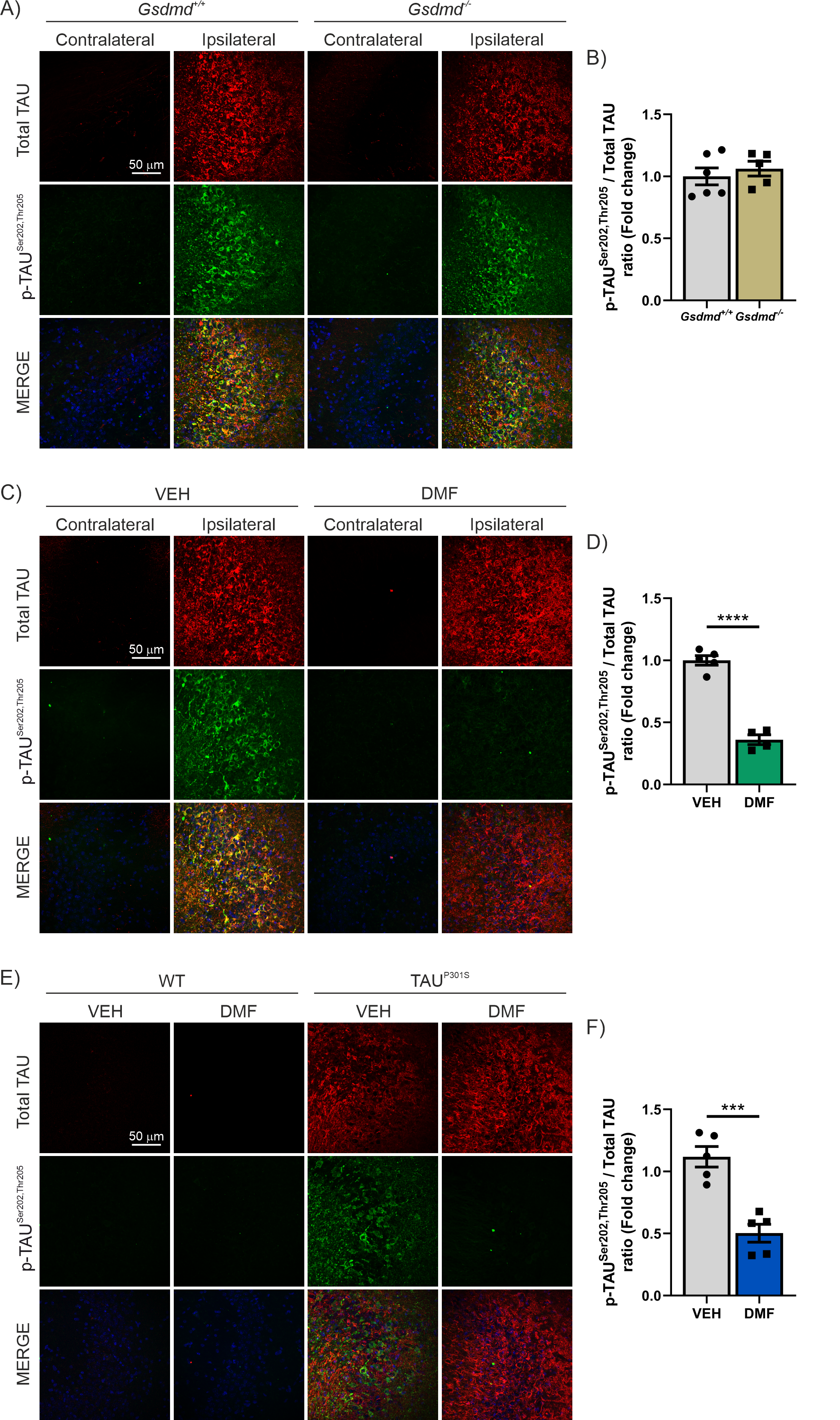

Supplement: Supplementary file 4 — Additional file 4 (Supplementary Figure 4. GSDMD deficiency does not alter TAU levels in AAV-TAUP301L mice and DMF treatment reduces TAU phosphorylation in hippocampus of both tauopathy mouse models. Immunostaining of total TAU (red) and p-TAUSer202,Thr205 (green) in the CA3 region of (A) Gsdmd+/+ and Gsdmd-/- mice that overexpress TAUP301L on the ipsilateral side, (C) AAV-TAUP301L mice treated with VEH or DMF and (E) 8-month-old Tg-TAUP301S mice and WT mice treated with VEH or DMF. Quantification of p-TAUSer202,Thr205/total TAU ratio of (B) Gsdmd+/+ and Gsdmd-/- mice that overexpress TAUP301L on the ipsilateral side, (D) AAV-TAUP301L mice treated with VEH or DMF and (F) 8-month-old Tg-TAUP301S mice and WT mice treated with VEH or DMF. Bars represent the mean of 4-6 samples ±SEM. Asterisks indicate significant differences of ***p<0.005; ****p<0.001 comparing each group by t-Student test.) [file 12929_2025_1210_MOESM4_ESM.tif]

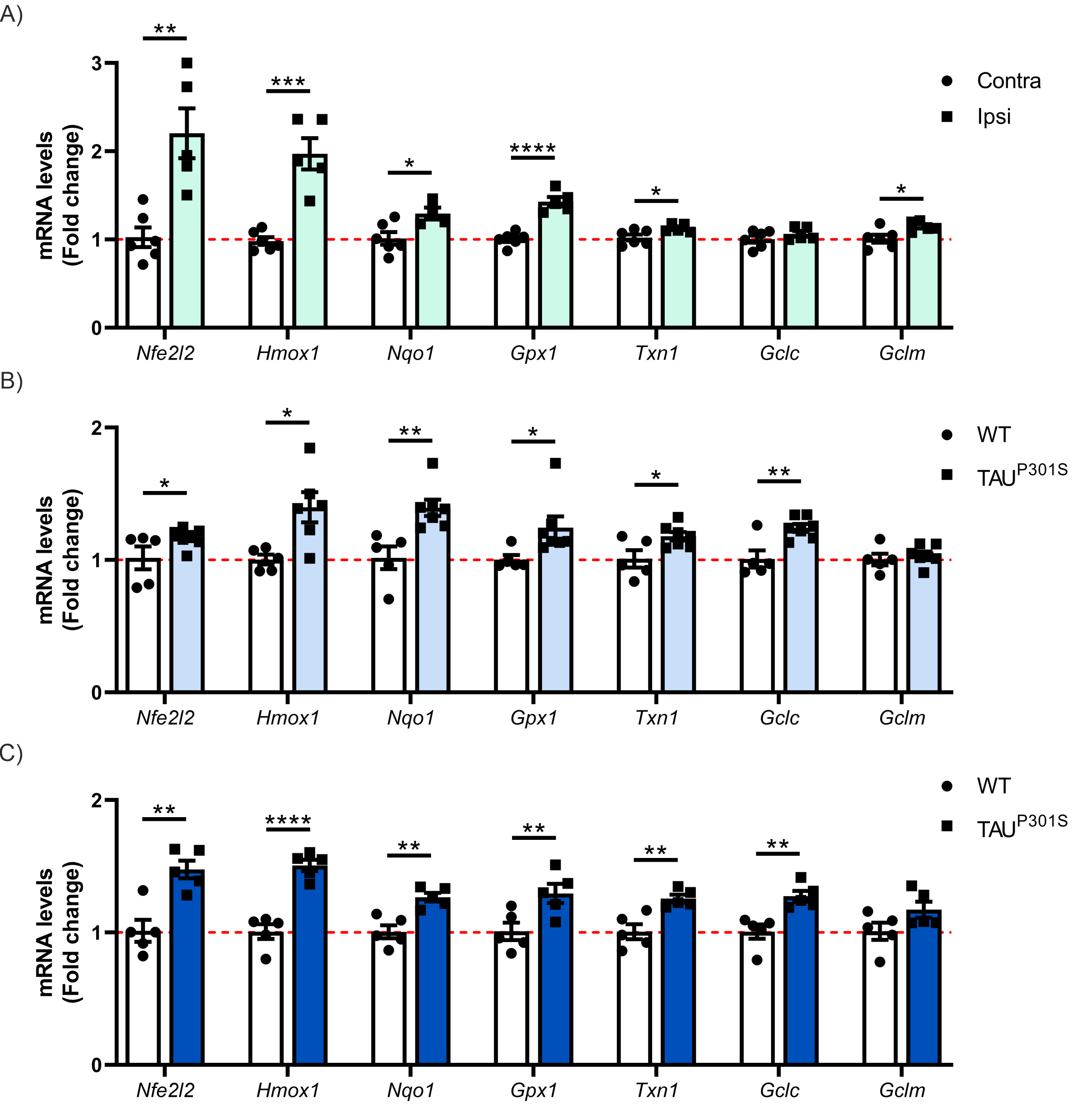

Supplement: Supplementary file 5 — Additional file 5 (Supplementary Figure 5. The overexpression of TAU induces NRF2 signaling pathway in hippocampus in both tauopathy mouse models. Analysis of NRF2-dependent enzyme mRNA levels in (A) AAV-TAUP301L mice (Contra: control side; Ipsi: TAU-overexpressing side); (B) 8-months-old and (C) 10-months-old transgenic TAUP301S mice. Bars represent the mean of 4-5 samples ±SEM. Asterisks indicate significant differences of *p<0.05; **p<0.01; ***p<0.005; ****p<0.001 comparing each group by t-Student test.) [file 12929_2025_1210_MOESM5_ESM.tif]

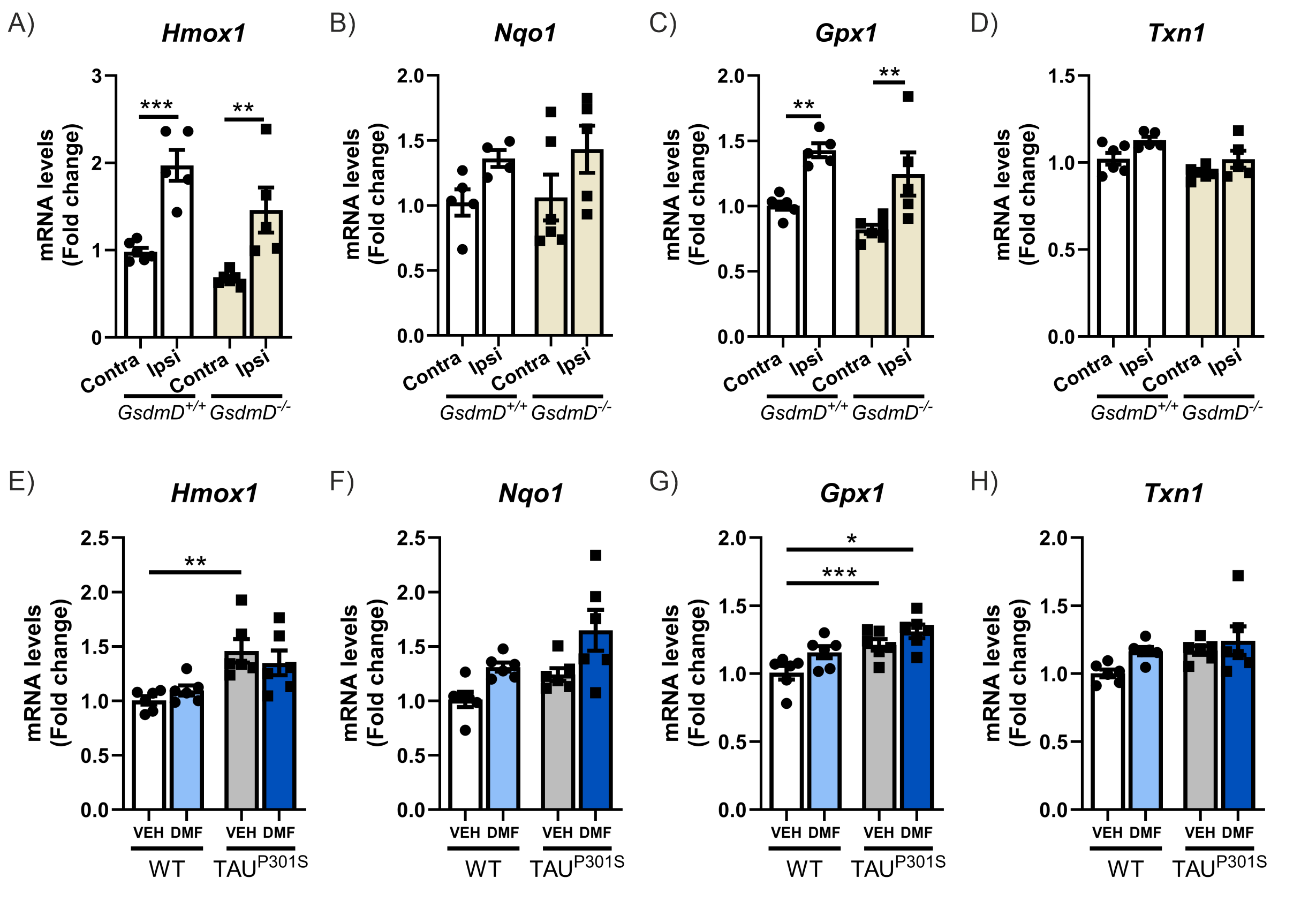

Supplement: Supplementary file 6 — Additional file 6 (Supplementary Figure 6. NRF2 signaling pathway in hippocampus is not altered by GSDMD deficiency and activated by DMF treatment. Analysis of mRNA levels of (A) Hmox1, (B) Nqo1, (C) Gpx1 and (D) Txn1 in the hippocampus of Gsdmd+/+ and Gsdmd-/- mice that overexpress TAUP301L on the ipsilateral side. Analysis of mRNA levels of (E) Hmox1, (F) Nqo1, (G) Gpx1 and (H) Txn1 in the hippocampus of 8-month-old Tg-TAUP301S mice and WT mice treated with VEH or DMF. Bars represent the mean of 4-5 samples ±SEM. Asterisks indicate significant differences of *p<0.05; **p<0.01; ***p<0.005 comparing each group by two-factor ANOVA test followed by Bonferroni post-test.) [file 12929_2025_1210_MOESM6_ESM.tif]

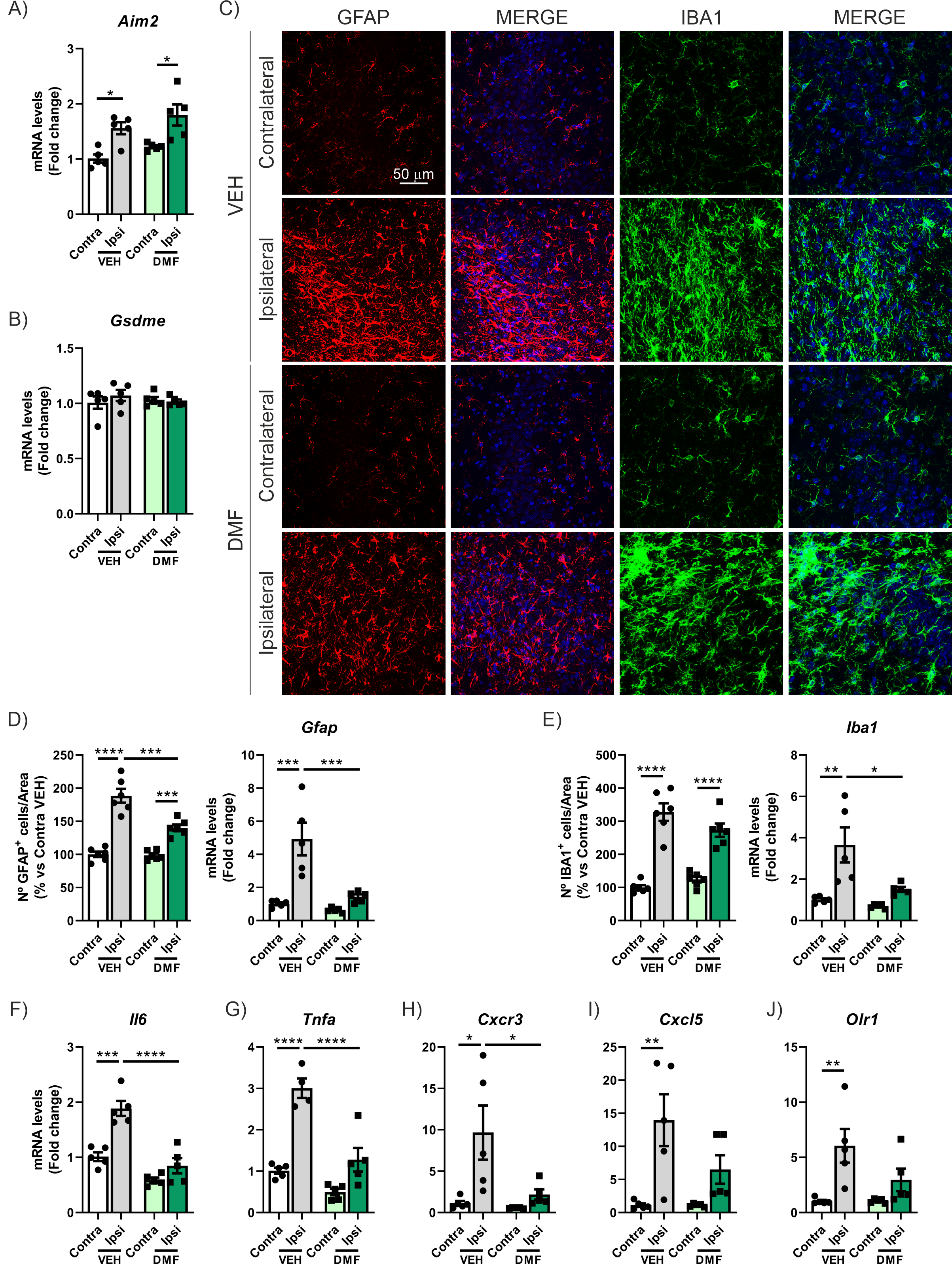

Supplement: Supplementary file 7 — Additional file 7 (Supplementary Figure 7. DMF treatment reduces the neuroinflammation in the hippocampus in the AAV-TAUP301L mouse model. Analysis of the mRNA levels of (A) Aim2 and (B) Gsdme in the hippocampus of AAV-TAUP301L mice treated with VEH or DMF. (C) Immunostaining of GFAP (red) and IBA1 (green) in the CA3 region. (D) Quantification of the number of GFAP+ cells and Gfap mRNA levels. (E) Quantification of the number of IBA1+ cells and Iba1 mRNA levels. Analysis of the mRNA levels of (F) Il6, (G) Tnfa, (H) Cxcr3, (I) Cxcl5 and (J) Olr1. Bars represent the mean of 4-5 samples ±SEM. Asterisks indicate significant differences of *p<0.05; **p<0.01; ***p<0.001; ****p<0.0001 comparing each group by two-factor ANOVA test followed by Bonferroni post-test.) [file 12929_2025_1210_MOESM7_ESM.tif]

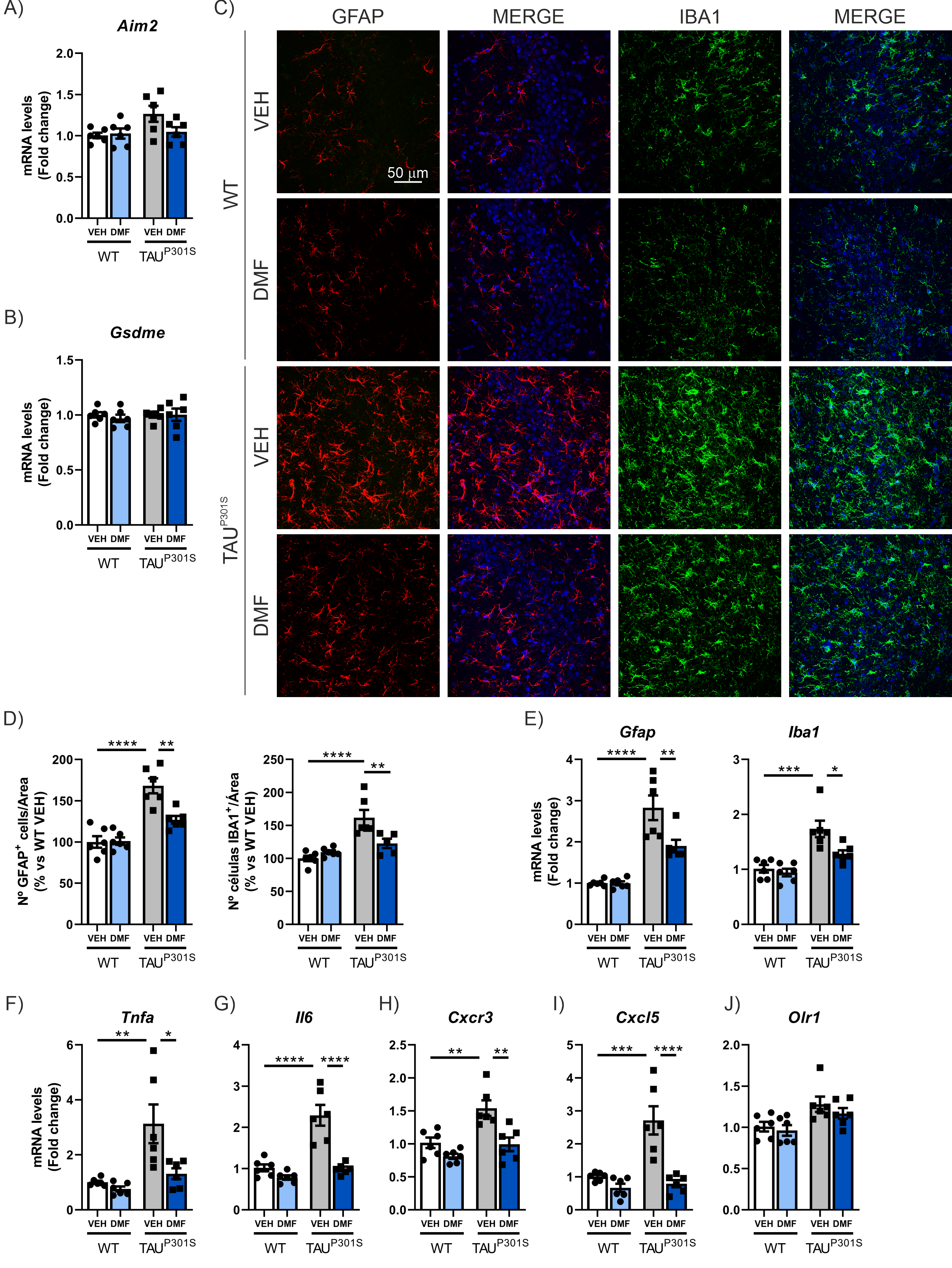

Supplement: Supplementary file 8 — Additional file 8 (Supplementary Figure 8. The induction of neuroinflammation markers is decreased by the treatment with DMF in the Tg-TAUP301S mouse model. Analysis of (A) Aim2 and (B) Gsdme mRNA levels in the hippocampus of 8-month-old Tg-TAUP301S mice and WT mice treated with VEH or DMF. (C) Immunostaining of GFAP (red) and IBA1 (green) in the CA3 region. (D) Quantification of the number of GFAP+ cells and Gfap mRNA levels. (E) Quantification of the number of IBA1+ cells and Iba1 mRNA levels. Analysis of the mRNA levels of (F) Il6, (G) Tnfa, (H) Cxcr3, (I) Cxcl5 and (J) Olr1. Bars represent the mean of 4-5 samples ±SEM. Asterisks indicate significant differences of *p<0.05; **p<0.01; ***p<0.001; ****p<0.0001 comparing each group by two-factor ANOVA test followed by Bonferroni post-test.) [file 12929_2025_1210_MOESM8_ESM.tif]

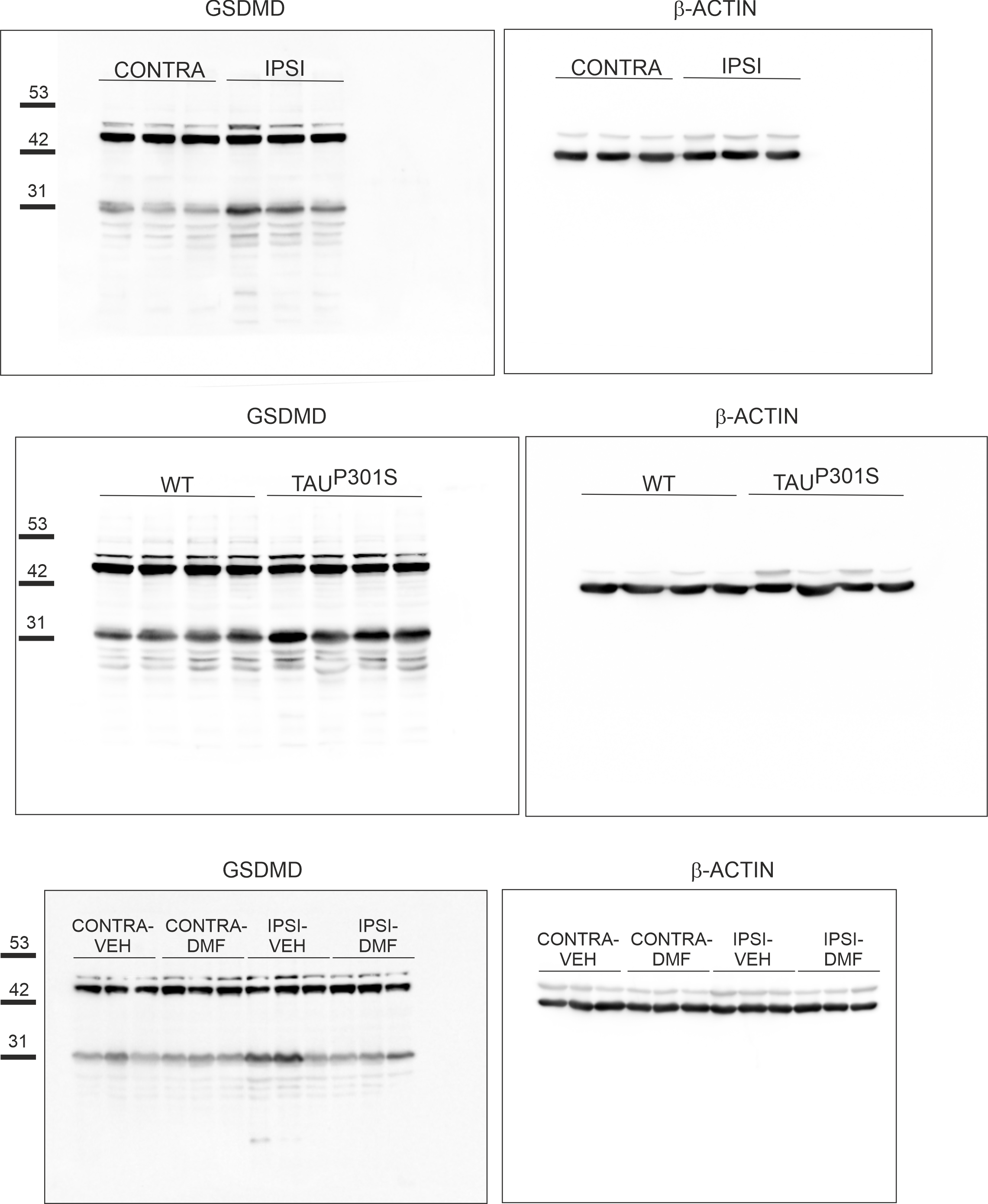

Supplement: Supplementary file 12 — Additional file 12. [file 12929_2025_1210_MOESM12_ESM.tif]
