# Supplementary material for: GASDERMIN D-mediated pyroptosis as a therapeutic target in TAU-dependent frontotemporal dementia mouse model
Source: J Biomed Sci. 2026 Jan 5;33:6. doi: 10.1186/s12929-025-01210-1 (PMC12766953; doi:10.1186/s12929-025-01210-1)
Supplement: Supplementary file 9 — Additional file 9. [file 12929_2025_1210_MOESM9_ESM.docx]

**Supplementary Table 1.** Characteristics of Alzheimer´s disease (AD) patients and controls (CTRL) used in this study.

| Experimental group | Code number | Gender | Age (years) |
| --- | --- | --- | --- |
| AD | BCPA 7 | Male | 80 |
| AD | BCPA 143 | Male | 88 |
| AD | BCPA 279 | Female | 98 |
| AD | BCPA 381 | Male | 86 |
| CTRL | BCPA 364 | Male | 43 |
| CTRL | BCPA 587 | Female | 83 |
| CTRL | BCPA 662 | Female | 58 |
