# Supplementary material for: GASDERMIN D-mediated pyroptosis as a therapeutic target in TAU-dependent frontotemporal dementia mouse model
Source: J Biomed Sci. 2026 Jan 5;33:6. doi: 10.1186/s12929-025-01210-1 (PMC12766953; doi:10.1186/s12929-025-01210-1)
Supplement: Supplementary file 10 — Additional file 10. [file 12929_2025_1210_MOESM10_ESM.docx]

**Table 2.** List of primers used in this study.

| **Gene Product** | **Forward Primer** | **Reverse Primer** |
| --- | --- | --- |
| *Actb* | 5’ TCCTTCCTGGGCATGGAG 3’ | 5’ AGGAGGAGCAATGATCTTGATCTT 3’ |
| *AIM2* | 5’ ATCTCCTGCTTGCCTTCTTGG 3’ | 5’ AAGTCTCTCCTCATGTTAAGCCTG 3’ |
| *Aim2* | 5’ GTCACCAGTTCCTCAGTTGTG 3’ | 5’ CACCTCCATTGTCCCTGTTTTAT 3’ |
| *ASC* | 5’ AGTGGCTGCTGGATGCTCTG 3’ | 5’ CATCTTGCTTGGGTTGGTGG 3’ |
| *Asc* | 5’ AGAGTACAGCCAGAACAGGA 3’ | 5’ GATGGAACAAAGCTGAAGAG 3’ |
| *Bdnf* | 5’ GATGCCGCAAACATGTCTATGA 3’ | 5’ TAATACTGTCACACACGCTCAGCTC 3’ |
| *CASP1* | 5’ TGAATACCAAGAACTGCCCAAG 3’ | 5’ GCATCATCCTCAAACTCTTCTGTAG 3’ |
| *Casp1* | 5’ GGAGAGAAACAAGGAGTGGTGT 3’ | 5’ ACTCAATGAAAAGTGAGCCCCT 3’ |
| *Cxcl5* | 5’ CCCTTCCTCAGTCATAGCCG 3’ | 5’ CTATGACTTCCACCGTAGGGC 3’ |
| *Cxcr3* | 5’ AGCCAAGCCATGTACCTTGAG 3’ | 5’ AGAAGTCGCTCTCGTTTTCCC 3’ |
| *Gapdh* | 5’ CGACTTCAACAGCAACTCCCACTCTTCC 3’ | 5’ TGGGTGGTCCAGGGTTTCTTACTCCTT 3’ |
| *Gclc* | 5’ TTACCGAGGCTACGTGTCAGAC 3’ | 5’ TATCGATGGTCAGGTCGATGTC 3’ |
| *Gclm* | 5’ AATCAGCCCCGATTTAGTCAGG 3’ | 5’ CCAGCTGTGCAACTCCAAGGAC 3’ |
| *Gfap* | 5’ TCCTGGAACAGCAAAACAAG 3’ | 5’ CAGCCTCAGGTTGGTTTCAT 3’ |
| *GSDMD* | 5’ CCCTCCTTCGAGCACTTC 3’ | 5’ TCTGGTGGTGTGTGCGTT 3’ |
| *Gsdmd* | 5’ TTCAGGGCAGAGTGATGTTGT 3’ | 5’ TCATCCCCACGACTCCGAA 3’ |
| *Gsdme* | 5’ AGTTTTCCTGGGGACTTGCTG 3’ | 5’ CGATGCCAAACCTCTCTGTGT 3’ |
| *Gpx1* | 5’ GGACTACACCGAGATGAACG 3’ | 5’ GATGTACTTGGGGTCGGTCA 3’ |
| *Hmox1* | 5’ CACAGATGGCGTCACTTCGTC 3’ | 5’ GTGAGGACCCACTGGAGGAG 3’ |
| *Iba1* | 5’ GTCCTTGAAGCGAATGCTGG3’ | 5’ CATTCTCAAGATGGCAGATC 3’ |
| *IL18RAP* | 5’ TGGGCTCTCAATTCCTTCTGT 3’ | 5’ AGCAGGGTCTCAGTTTCAGC 3’ |
| *Il18rap* | 5’ GTCCGAGCTGTGGTTAAAGTG 3’ | 5’ AGGCTTTCCAAGCTCTACGTC 3’ |
| *IL1B* | 5’ AGCTCGCCAGTGAAATGATG 3’ | 5’ TCGGAGATTCGTAGCTGGATG 3’ |
| *Il1b* | 5’ CTGGTGTGTGACGTTCCCATTA 3’ | 5’ CCGACAGCACGAGGCTTT 3’ |
| *Il6* | 5’ CCTACCCCAATTTCCAATGCT 3’ | 5’ TATTTTCTGACCACAGTGAGGAATG 3’ |
| *MAPT* | 5’ GTCCAAGTGTGGCTCAAAG 3’ | 5’ CTGGTTTATGATGGATGTTGC 3’ |
| *Nfe2l2* | 5’ CCCGAAGCACCCTGAAGGCA 3’ | 5’ CCAGGCGGTGGGTCTCCGTA 3’ |
| *NLRP3* | 5’ AGCCCCGTGAGTCCCATTA 3’ | 5’ ACGCCCAGTCCAACATCATCT 3’ |
| *Nlrp3* | 5’ CCCTTGGAGACACAGGACTC 3’ | 5’ GAGGCTGCAGTTGTCTAATTCC 3’ |
| *Nqo1* | 5’ GGTAGCGGCTCCATGTACTC 3’ | 5’ CATCCTTCCAGGATCTGCAT 3’ |
| *Olr1* | 5’ AGCAGGAATTTGGAGATGACTTT 3’ | 5’ GTCATAGCAGCAGGGAACCA 3’ |
| *Tbp* | 5’ TGCACAGGAGCCAAGAGTGAA 3’ | 5’ CACATCACAGCTCCCCACCA 3’ |
| *Tnfa* | 5’ CATCTTCTCAAAATTCGAGTGACAA 3’ | 5’ TGGGAGTAGACAAGGTACAACCC 3’ |
| *Trpc6* | 5’ CTTCGGCCGTCCAAATCTCA 3’ | 5’ CTGCTGCCGTAAACCAGAGA 3’ |
| *Txn1* | 5’ CTCCCCGCAACAGCCAAAAT 3’ | 5’ CAGAGAAGTCCACCACGACAA 3’ |
