# Supplementary material for: GASDERMIN D-mediated pyroptosis as a therapeutic target in TAU-dependent frontotemporal dementia mouse model
Source: J Biomed Sci. 2026 Jan 5;33:6. doi: 10.1186/s12929-025-01210-1 (PMC12766953; doi:10.1186/s12929-025-01210-1)
Supplement: Supplementary file 11 — Additional file 11. [file 12929_2025_1210_MOESM11_ESM.docx]

**Supplementary Table 3.** List of antibodies used in this study.

| Antibody | Source | Catalog Number | Dilution |
| --- | --- | --- | --- |
| Alexa Fluor 488 donkey anti-mouse IgG | Thermo Fisher Scientific | A21202 | 1:500 IF |
| Alexa Fluor 488 donkey anti-rabbit IgG | Thermo Fisher Scientific | A21206 | 1:500 IF |
| Alexa Fluor 555 donkey anti-mouse IgG | Thermo Fisher Scientific | A31570 | 1:500 IF |
| Alexa Fluor 555 donkey anti-rabbit IgG | Thermo Fisher Scientific | A31572 | 1:500 IF |
| Alexa Fluor 488 goat anti-guinea pig IgG | Invitrogen | A11073 | 1:500 IF |
| BDNF | Santa Cruz Biotechnologies | sc-20981 | 1:500 IF |
| CALBINDIN-D28K | Synaptic Systems | 214.002 | 1:500 IF |
| GASDERMIN D | Abcam | ab219800 | 1:500 IF  1:1000 WB |
| GFAP | Sigma-Aldrich | G3893 | 1:400 IF |
| IBA1 | Wako Chemicals | 019-19741 | 1:500 IF |
| IBA1 | Synaptic Systems | HS-234 308 | 1:1000 IF |
| p-TAU^Ser202/Thr205^ (AT8) | Thermo Fisher Scientific | #MN1020 | 1:1000 IF |
| Total human TAU | Santa Cruz Biotechnologies  ThermoFisher Scientific | sc-5587  #MN1000B | 1:200 IF  1:100 IHC/IF |
